# Molecular dynamics study of the internalization of cell-penetrating peptides containing unnatural amino acids across membranes†

**DOI:** 10.1039/d1na00674f

**Published:** 2021-11-10

**Authors:** Joan Gimenez-Dejoz, Keiji Numata

**Affiliations:** Biomacromolecules Research Team, RIKEN Center for Sustainable Resource Science Saitama Japan; Department of Material Chemistry, Graduate School of Engineering, Kyoto University Kyoto Japan numata.keiji.3n@kyoto-u.ac.jp

## Abstract

Peptide-based delivery systems that deliver target molecules into cells have been gaining traction. These systems need cell-penetrating peptides (CPPs), which have the remarkable ability to penetrate into biological membranes and help internalize different cargoes into cells through the cell membranes. The molecular internalization mechanism and structure–function relationships of CPPs are not clear, although the incorporation of nonproteinogenic amino acids such as α-aminoisobutyric acid (Aib) has been reported to increase their helicity, biostability and penetration efficiencies. Here, we used molecular dynamics to study two Aib-containing CPPs, poly(LysAibAla)_3_ (KAibA) and poly(LysAibGly)_3_ (KAibG), that previously showed high cell internalization efficiency. KAibA and KAibG displayed the lowest internalization energies among the studied CPPs, showing distinct internalization mechanisms depending on the lipid composition of the model membranes. The presence of Aib residues allows these CPPs to adopt amphipathic folding to efficiently penetrate through the membranes. Elucidating how Aib incorporation affects CPP–membrane binding and interactions is beneficial for the design of CPPs for efficient intracellular delivery.

## Abstract 2

Peptide-based delivery systems that deliver target molecules into cells have been gaining traction.

## Graphical abstract

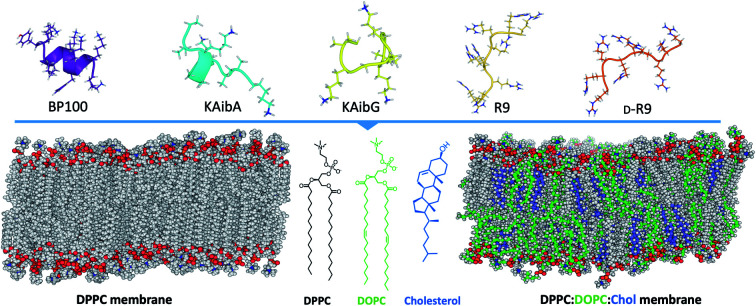

## Introduction

Cell-penetrating peptides (CPPs) are normally short cationic or/and amphiphilic peptides that have the ability to penetrate biological membranes.^1^ Due to their high affinity for membranes, CPPs have been used as delivery agents to carry a wide range of biomolecules into cells. Moreover, they can be specifically modified with targeting sequences to achieve targeted delivery of the cargo into subcellular components such as specific organelles.^2–7^ However, to increase the use of CPPs as delivery systems, a series of problems must be overcome. Peptide carriers have to be stable enough to maintain their structure in a complex environment, internalize into cells, perform lysosomal escape and target a specific organelle to release their cargo. Thus, most CPP research is aimed at increasing the biostability, penetration and specificity of CPPs.

Several studies have investigated the structure–activity relationship of CPPs, including how their folding, hydrophobicity, and net charge affect their function.^8^ Despite the discovery of thousands of CPPs that vary widely in the number of residues, sequence and secondary structure,^9^ their internalization mechanism remains controversial, and there is no clear understanding of the molecular mechanisms underlying their dynamic behaviors of membrane binding and penetration.^10^ The main characteristics that increase the penetration of CPPs are their charge and the adoption of a helical amphiphilic structure.^11–13^ Helicity has been related to the higher antimicrobial activity of some peptides,^14,15^ and most transmembrane domains of proteins are α-helical.^16^ Helical structures effectively shield the polarity of the oxygen and nitrogen atoms in the protein backbone that form intramolecular hydrogen bonds, making this secondary structure energetically preferred.^17^ Thus, the construction or modification of peptides with helical structures, with cationic residues on one side of the helix and hydrophobic residues on the other side, is expected to increase the internalization efficiency of CPPs.

Peptides with higher biostability have longer circulation times and can achieve higher steady-state concentrations, thus increasing their usefulness as therapeutic agents and delivery systems.^18^ The basic problem is that CPPs are easily recognized by proteases and are susceptible to enzymatic degradation.^19,20^ One solution is the incorporation of nonproteinogenic amino acids, such α-aminoisobutyric acid (Aib), or d-amino acids into peptides, which changes the secondary structural patterns induced by l-amino acids that are widely recognized by proteases.^6,13,21^ Moreover, these other amino acids can form backbone angles that are unfavorable for l-amino acids and can thus stabilize helical structures,^22^ as in the case of Aib, an achiral but conformationally constrained residue often used as a helix inducer.^23,24^ Previously, a positive correlation has been observed between the helical structures and cell penetrating ability of Aib-incorporated CPPs.^12^ Thus, its incorporation into CPPs is advantageous because it serves a dual purpose: on the one hand, it increases the helical conformation and penetration ability of peptides, and on the other hand, it increases the biostability of the peptides, conferring intrinsic resistance to proteases.^6^

**Fig. 1:**
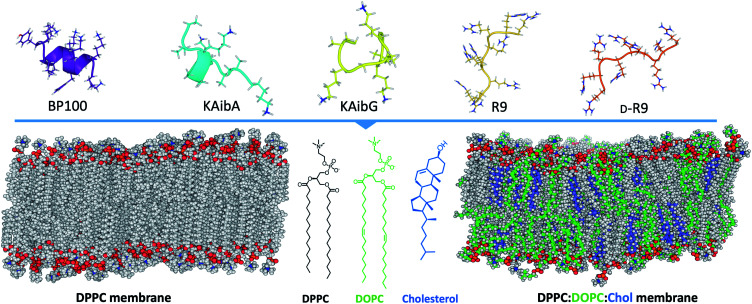
Schematic representation of the systems investigated in this study, showing the structures of the peptides employed and the two membrane systems employed. DPPC; dipalmitoylphosphatidylcholine, DOPC; dioleoylphosphatidylcholine, R_9_; nona-arginine, d-R_9_; nona-arginine composed of d-amino acids, KAibA; poly(LysAibAla)_3_, and KAibG; poly(LysAibGly)_3_. DPPC molecules are shown in gray in the membranes, DOPC in green and cholesterol in blue.

Here, we investigate the interaction and internalization of Aib-incorporated CPPs into membranes using molecular dynamics (MD) simulations with enhanced sampling techniques. We studied poly(LysAibAla)_3_ (KAibA) and poly(LysAibGly)_3_ KAibG, two recently designed helical peptides incorporating Aib residues that exhibit improved biostability and internalization abilities in human and plant cells.^6,25^ Moreover, widely known CPPs with distinct structures, such as helical BP100,^3,26,27^ and nonhelical nona-arginine (R_9_)^28,29^ and its chiral peptide d-R_9_, were modeled and compared with KAibA/G (Fig. 1). We used two model membranes, one composed of dipalmitoyl phosphatidylcholine (DPPC), which is commonly used to prepare liposomes for drug delivery and to investigate membrane stability and permeability in biophysical studies,^30^ and a second model composed of dipalmitoyl phosphatidylcholine (DPPC), dioleoyl phosphatidylcholine (DOPC) and cholesterol (Chol) (1 : 1 : 1) (DPPC : DOPC : Chol) to more accurately model the plasma membrane (Fig. 1).^31,32^ The comparison of peptide internalization energies and the dynamic behavior of the CPPs in the membrane with knowledge of local peptide–lipid interactions might prove helpful to understand the internalization mechanism of peptides into cells and to compare the internalization efficiency of CPPs.

## Materials and methods

### Peptide construction and folding

The initial folded structures of the poly(LysAibAla) and poly(LysAibGly) peptides were taken from a previous study.^6^ The BP100 peptide^33^ (Lys-Lys-Leu-Phe-Lys-Lys-Ile-Leu-Lys-Tyr-Leu) was modeled with an α-helix conformation, whereas the random coiled R_9_ peptide (Arg-Arg-Arg-Arg-Arg-Arg-Arg-Arg-Arg)^34^ and d-R_9_ chiral form were constructed with a linear conformation with the tleap module of Ambertools.^35^ The peptides were modeled with the AMBER ff14SB force field,^36^ and both systems were solvated with TIP3P water molecules and counterions.^37^ Periodic boundary conditions were applied to the systems, and the SHAKE algorithm^38^ was used to restrain the bonds involving hydrogen atoms, allowing a time step of 2 fs for the simulations. Temperature and pressure were coupled to an external bath.^39^ Particle mesh Ewald (PME) summation^40,41^ was used to calculate the electrostatic interactions, with a cutoff of 8.0 Å for long-range interactions. The systems were energy-minimized for 20 000 steps using the conjugate gradient minimization algorithm and then slowly heated to 300 K over 600 ps with the solute atoms fixed with a 2 kcal mol^−1^ restraint. Then, the system was equilibrated in the NPT ensemble over 500 ps to ensure the appropriate pressure and density of the water box with the Berendsen barostat, the restraints were removed, and the peptides were equilibrated for 100 ns.

### Construction of DPPC and DPPC : DOPC : Chol membranes

We constructed two different model membrane systems for this work using the CHARMM-GUI Membrane Builder.^42–46^ The first consisted of a model membrane composed of only dipalmitoylphosphatidylcholine (DPPC) lipids, and the other model was composed of dipalmitoylphosphatidylcholine (DPPC), dioleoylphosphatidylcholine (DOPC) and cholesterol (Chol) in a ratio of 1 : 1 : 1 ((DPPC : DOPC : Chol)). The systems were created using a rectangular box type with 300 lipid components in both the upper and lower leaflets; 150 mM KCl ions were added using the Monte Carlo method, and TIP3P water molecules were added.

These systems of peptides and membranes were then created with tleap using the amber lipid14 forcefield^47,48^ for lipids. To construct these peptide and membrane systems, the folded peptides were positioned at approx. 10 Å on the membrane surface. We used the same equilibration procedures as described for the folding of the peptides, with temperature equilibration in the NVT and pressure equilibration in the NPT ensembles, except that the restraint on the solute atoms of the system was 10 kcal mol^−1^ and heating was carried out for 1 ns. The systems were equilibrated for at least 100 ns with a distance restraint to prevent the peptide from coming into contact with the membrane, thus allowing the water and membrane to equilibrate before starting productive simulations.

### Steered molecular dynamics and Jarzynski equality

Steered molecular dynamics^49,50^ (SMD) is an enhanced sampling technique that applies an external force on a biomolecular system and leads to a change in its coordinates over time that can be used to calculate the potential of mean force (PMF) along a pulling coordinate.^51–53^ For the SMD simulations, we computed the work using the following approach: we defined a pulling coordinate in the normal (*z*) direction (perpendicular to the membrane surface) from the center of mass (COM) of the main chain atoms of the peptides to the COM of the atoms forming the opposite site of the bilayer. For the DPPC membrane, we used the P, N, C1, C2 and C3 atoms of the lipids, and for the DOPC membrane, we used the P, C2, N, and PC atoms of the DPPC and DOPC lipids and the O1 atom of the cholesterol membrane. We used two different harmonic restraints of 1 and 10 kcal mol^−1^ for all peptides and simulations, and the peptides were pulled from the top bilayer (*z* ∼ 55 Å) to the bottom bilayer (*z* = 0 Å). We used a constant pulling velocity of 1 Å ns^−1^, similar to those used in previous studies.^50,54^ Due to the large distance of the coordinate traversing the bilayer, we used the adaptive steered molecular dynamics technique^55–57^ and divided the length of the coordinate into different stages of 5 Å, computing 50 replicas for each stage. The obtained work for each replica (obtained as the integrated force over distance) was used to calculate the free energy using the Jarzynski equality.^50,58,59^ We selected the replica that presents the work closest to the Jarzynski average and used it to initiate the next stage using SMD. This accounted for 500 to 550 replicas for each system, for a total accumulated simulation time of ∼2.75 μs.

### Adaptively biased molecular dynamics

We used the enhanced sampling technique adaptively biased molecular dynamics (ABMD)^60^ with a well-tempered algorithm^61^ to determine the free-energy landscapes (FELs) of the peptides crossing the membranes. ABMD is a technique based on metadynamics^62^ in which a time-dependent biasing potential is introduced over selected collective variables (CVs) and compensates for the underlying FEL. This effectively discourages the system from returning to the previously sampled space and forces it to explore regions of high free energy. We used ABMD together with the well-tempered algorithm^61^ and the multiple-walker scheme,^63^ with 8 replicas per system. The simulated systems were the same as those used for SMD, and 8 replicas from the initial SMD conformations were randomly selected for each system as starting walkers. We used one CV, consisting of the distance between the COM of the main chain atoms of the peptide and the COM of the atoms forming the opposite site of the bilayer, as described previously for SMD. A flooding timescale of 40 ps for the bias deposition (20 000 MD steps) with a resolution of 0.5 kcal mol^−1^ (for KAibA and KAibG) and a flooding timescale of 10 ps for the bias deposition (5000 MD steps) with a resolution of 1.0 kcal mol^−1^ (for BP100, R_9_, d-R_9_, KAibA and KAibG fast simulations) were used. The simulations were processed and analyzed using CPPTRAJ,^64^ the graphics were plotted with GNUplot or python Matplotlib library,^65^ and the molecular structures were visualized with VMD software^66^ and PyMol (PyMOL Molecular Graphics System, v 1.8, Schrödinger).

## Results and discussion

### Steered molecular dynamics

We first calculated the PMF of the KAibA and KAibG peptides internalized into the DPPC membrane using SMD and 2 different pulling harmonic forces, 1 and 10 kcal mol^−1^. We observed higher variability between the energies obtained with the peptides crossing the DPPC membrane than between those obtained with the peptides crossing the DPPC : DOPC : Chol membrane (Table 1, Fig. S1 and S2†). The secondary structures of the peptides were not altered significantly when the pulling harmonic force was changed from 1 kcal mol^−1^ to 10 kcal mol^−1^, with the exception of KAibA in the DPPC membrane (Fig. S3 and S4†).^67^

**Table 1:** Energies for the internalization of the KAibA and KAibG peptides into the DPPC membrane and DPPC : COPC : Chol membrane obtained by SMD using 2 harmonic pulling forces

Membrane	Peptide	Harmonic force (kcal mol^−1^)	PMF (kcal mol^−1^)
DPPC	KAibA	1	106.4
		10	144.8
DPPC	KAibG	1	152.3
		10	78.3
DPPC : DOPC : Chol	KAibA	1	141.4
		10	135.5
DPPC : DOPC : Chol	KAibG	1	145.2
		10	157.5

The simulation of KAibG in the DPPC membrane with a pulling force of 10 kcal mol^−1^ presented the lowest energy, with a value of 78.3 kcal mol^−1^ (Table 1, Fig. S1†), with KAibG mainly adopting a bend-and-turn conformation (Fig. S3b†). The carboxyl group of the C-terminal glycine forms polar contacts with the Gly3 main chain nitrogen atom and the amine group of the N-terminal Gly, keeping the C-terminus locked during all the simulations. This gives the peptide a compact structure, shields the polar group and keeps all Aib hydrophobic residues on one side and the cationic Lys residues on the opposite, creating an amphiphilic peptide. The peptide penetrates the membrane with its hydrophobic face toward its interior, keeping its lysine residues oriented toward phosphatidylcholine (PC) located on the upper membrane leaflet. The same secondary structural patterns of KAibG were observed in the simulation with a harmonic force of 1 kcal mol^−1^ and in those performed later with ABMD. In contrast, in the DPPC : DOPC : Chol membrane, the peptide retains the bend propensity at Gly3, but this glycine does not come into contact with the N-terminal amine of the peptide. Here, KAibG presents a propensity increase at residues 5 to 7 (Aib, Gly and Lys) compatible with a 3_10_-helix secondary structure (Fig. S4†), which forms strong intramolecular H-bonds. KAibA mainly presents a mixture of α- and 3_10_-helical conformations, in agreement with previous studies,^6,23^ adopting only a turn conformation with a force constant of 1 kcal mol^−1^ in the DPPC membrane. It internalizes into the membrane with a helical structure, orienting its Ala and Aib residues toward the interior while keeping Lys in contact with the PC groups.

The peptides in the DPPC membrane presented generally lower internalization energies (the calculated free energy that is required for moving from one side of the membrane to the opposite leaflet) than those in the DPPC : DOPC : Chol membrane, and the DPPC membrane presented more disturbance and thinning before peptide internalization. SMD is a technique that applies an external pulling force to the system; thus, some energies obtained could be artifacts of the force applied or poor convergence. The high variability between the energies obtained for the DPPC membrane compared with the energies obtained for the DPPC : DOPC : Chol membrane seems to suggest the presence of such artifacts. Even at the slow pulling velocity used here (1 Å ns^−1^), it is possible that the peptides did not have enough time to adapt to their environment and maintained a similar structure to their initial folding. To overcome these limitations, we simulated the systems using ABMD,^60^ a metadynamics-based method,^62^ with the well-tempered algorithm.^61^

### Adaptively biased MD KAibA and KAibG in the DPPC membrane

We performed ABMD simulations with KAibA and KAibG in the DPPC membrane, and the obtained energies for their penetration are shown in Table 2. The internalization of the peptides into the DPPC membrane presented a lower energy than that in the more complex DPPC : DOPC : Chol membrane, consistent with the SMD simulations. Likewise, the lowest internalization energy corresponded to the KAibG peptide, which presents an energy of 52.3 kcal mol^−1^ for membrane crossing, followed by KAibA, with an energy of 75.9 kcal mol^−1^ (Fig. S5,†Table 2).

**Table 2:** Energies obtained by ABMD for the internalization of the KAibA and KAibG peptides into the DPPC and DPPC : COPC : Chol membranes

Membrane	Peptide	Δ*G* (kcal mol^−1^)
DPPC	KAibA	75.9
	KAibG	52.3
DPPC : DOPC : Chol	KAibA	101.8
	KAibG	104.8

**Fig. 2:**
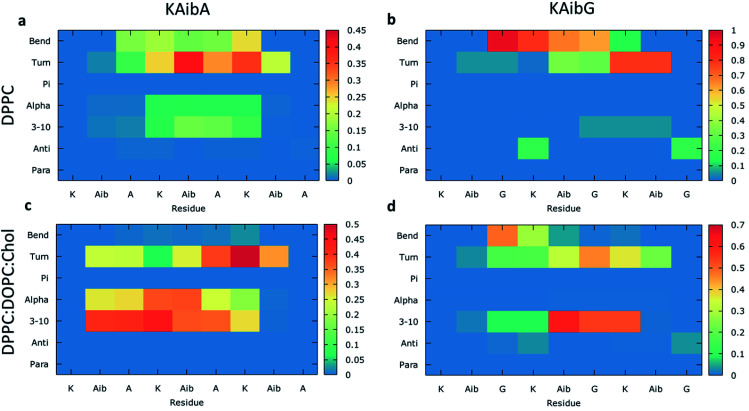
Average structural propensities of the KAibA and KAibG peptides in the DPPC (a and b) and DPPC : DOPC : Chol membranes (c and d) calculated over all trajectories for each amino acid residue. Para: parallel beta-sheet. Anti: anti-parallel beta-sheet. 3–10: 3–10 helix. Alpha: alpha-helix. Pi : Pi (3–14) helix. The color bar indicates propensity.

**Fig. 3:**
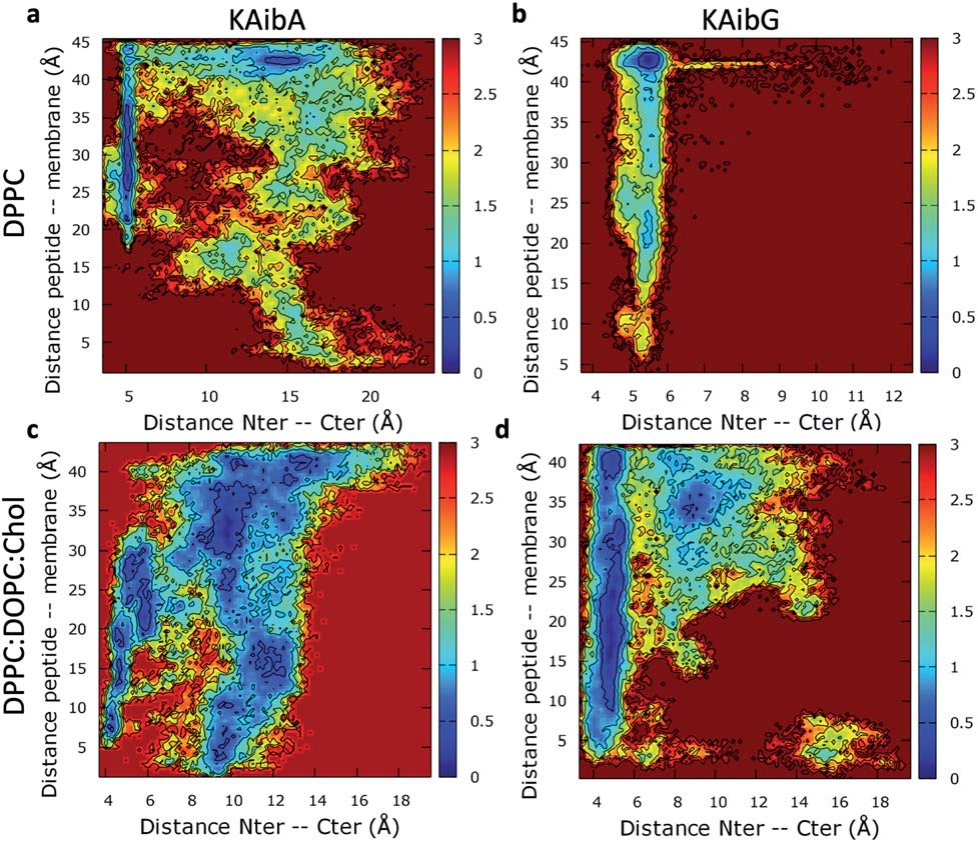
Two-dimensional histograms of the KAibA and KAibG peptides on the DPPC (a and b) and DPPC : DOPC : Chol (c and d) membranes from the COM distance between the α-carbon of the N-terminal and C-terminal amino acids of the peptides, and the COM distance of the peptides to the membrane was used as a CV for the ABMD simulations. The free energy (kcal mol^−1^) was estimated based on the bin population at 300 K for all frames for each amino acid residue.

**Fig. 4:**
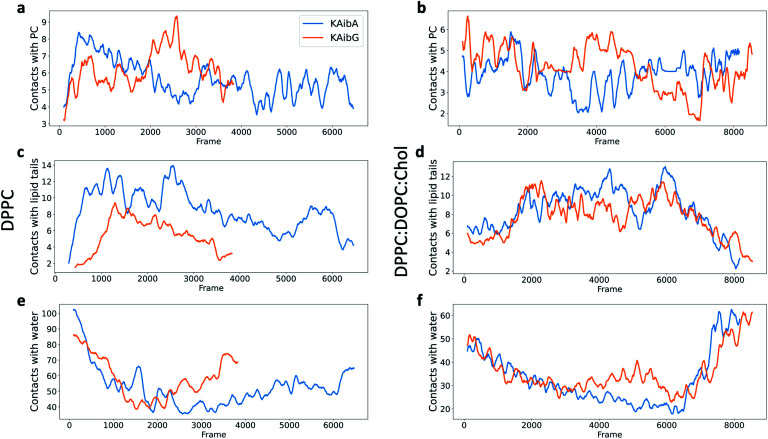
Number of contacts (water molecules located at less than 3.5 Å) between KAibA or KAibG with phosphatidylcholine (PC) (a and b), lipid tails (c and d) or water (e and f) over the simulation time. The DPPC membrane (a, c, and e). The DPPC : DOPC : Chol membrane (b, d, and f). The lipid tails are palmitoyl groups for the DPPC membrane (c) and palmitoyl plus oleoyl for the DPPC : DOPC : Chol membrane (d). The data are a rolling average over 100 data points.

We analyzed the secondary structure of the peptides by calculating their propensities (Fig. 2) and the structural changes during membrane internalization by examining the COM distance between the N-terminus and C-terminus of the peptides over the trajectory ensemble (Fig. 3). KAibG presented a more defined structure than KAibA in both membranes, as seen from the secondary structural propensity values (Fig. 2). In the DPPC membrane, Gly3 is locked in a bent conformation, while in the DPPC : DOPC : Chol membrane Aib5, Gly6 and Lys7 residues prefer a 3_10_- or α-helix conformation. These are the same secondary structural propensities observed previously using SMD. Moreover, KAibG presents its N- and C-termini at short distances during internalization (Fig. 3b) because its C-terminus is in contact with the amino group of the N-terminus and the N atom of Gly3, effectively shielding its polar groups and resulting in a compact structure. When the N-terminus and C-terminus of KAibG are dissociated, the peptide does not penetrate the membrane (Fig. 3b). KAibG internalizes with its hydrophobic residues (Aib) oriented toward the inside of the membrane and its Lys residue is in contact with the PC groups of the upper leaflet, pushing these groups inside to maintain a high number of contacts during all simulations (Fig. 4a) until one Lys residue comes into contact with those in the lower leaflet. The KAibA secondary structural propensity matched that observed with SMD using a 1 kcal mol^−1^ harmonic force. However, it has a lower tendency to form a secondary structure (Fig. 2a), with a lower propensity value than that obtained by SMD (Fig. 2a and S3a). KAibA is able to adopt different structural configurations on the surface of the membrane, with a favorable configuration when its terminus is located at 15 Å (Fig. 3a). When it internalizes into the membrane, it generally adopts a more compact structure, shielding its charged terminus from the hydrophobic lipid tails, and its Ala residues orient toward the hydrophobic membrane interior. However, when located close to the PC groups of the lower leaflet (Fig. 3), its N- and C-termini separate, and its Lys reaches the lower leaflet PC groups.

KAibG presents a lower internalization energy than KAibA, which is attributed mainly to its smaller and more compact structure when penetrating into the membrane. Both peptides display a similar number of contacts with the PC groups of the lipids (Fig. 4a), although KAibA shows slightly fewer contacts and a higher number of PCs (Fig. S6a†). This finding correlates with the slightly lower number of H-bonds that KAibG forms during the simulation compared with KAibA (Table S1 and Fig. S7†). KAibA presents a higher number of nearby PCs at the start of the simulation (Fig. 4a) before penetrating the membrane, while KAibG shows fewer contacts. Likewise, it has a higher number of close palmitoyl (PA) molecules (lipid tails) than KAibG (Fig. 4c) and forms more contacts with a larger number of lipid tails (Fig. S6c†), suggesting a stronger hydrophobic interaction with the lipid tails and its hydrophobic groups. This finding could explain the lower internalization energy displayed by KAibG, as it has a compact structure and forms fewer hydrophobic interactions with the lipid tails (Fig. 4c) while still being able to bind and interact with the PC groups.

**Fig. 5:**
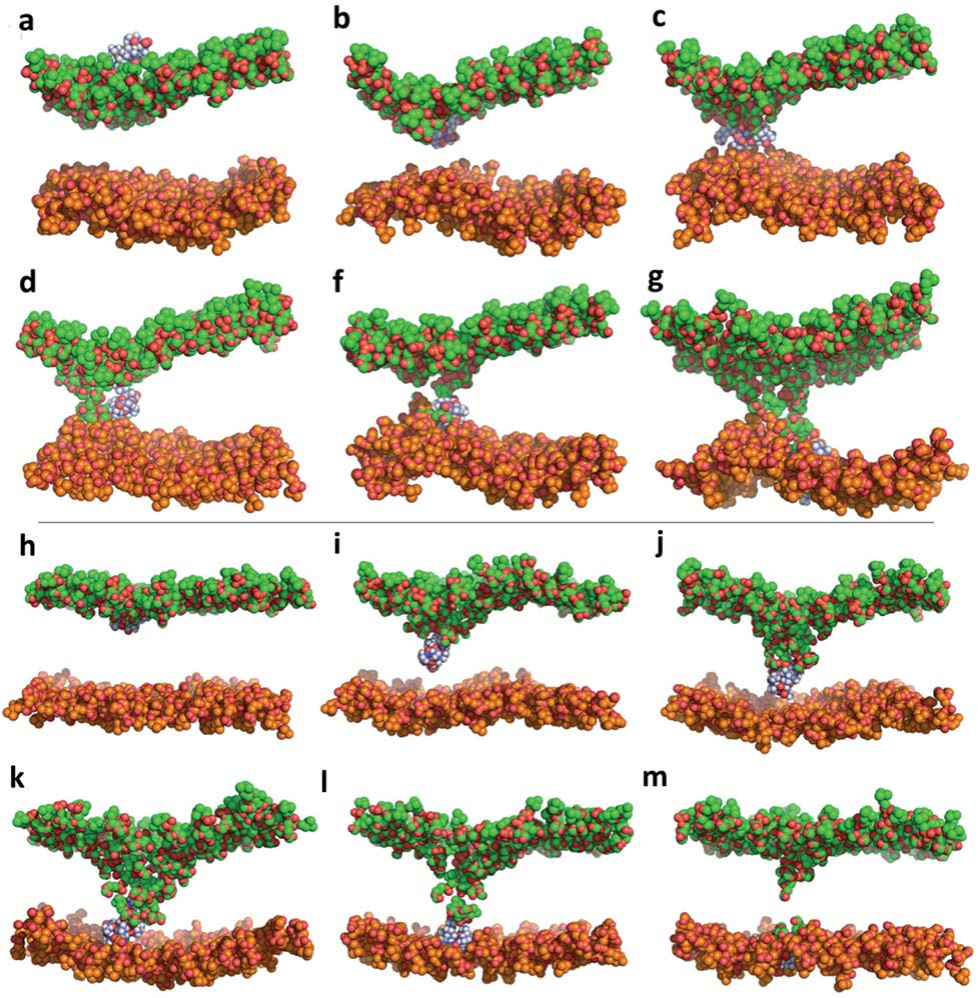
Molecular dynamics simulation of KAibA internalization into the DPPC membrane (a–g) and the DPPC : DOPC : Chol membrane (h–m). Only the PC groups of the lipids are shown for clarity. The carbon atoms of the upper leaflet and lower leaflet of the membrane are shown in green and orange, respectively. Peptide carbon atoms are shown in blue.

Both KAibA and KAibG resulted in thinning and disruption of the DPPC membrane before penetrating it. The upper and lower leaflets of the PC groups were not separated by the lipid tails of both leaflets facing each other in the thinning region; instead, the lipid tails of the upper and lower leaflets were mixed in one layer, making the membrane considerably thinner and increasing its bending (Fig. 5). Membrane disruption of the DPPC membrane was also observed, although less prominently, in the SMD simulation. Both peptides penetrated the membrane, with their hydrophobic amino acids turned toward the inside of the membrane, and the Lys residues oriented toward the PC groups of the outer membrane leaflet, pushing them toward the inside of the membrane (Fig. 5b, c, S9). Due to membrane thinning, the PC groups of the lower leaflet were closer to those in the upper leaflet (Fig. 5c–f) until the PC of both leaflets finally was in contact with the peptide. The PC groups effectively connected both sides of the membrane, creating a pore through which water molecules could pass (Fig. 5). The passage of water molecules together with the peptides is reflected in the number of close water molecules surrounding the peptides, showing a decrease during internalization in the hydrophobic membrane (Fig. 4e, S6a†) and recovery in the last step of the simulation as the peptides come into contact with the opposite side. In addition, there were more contacts with water in the DPPC membrane than in the DPPC : DOPC : Chol membrane (Fig. 4e, f and S8†).

### Adaptively biased MD with KAibA and KAibG in the DPPC : DOPC : Chol membrane

The energies required for KAibA and KAibG to penetrate the DPPC : DOPC : Chol membrane were similar, with values 101.8 kcal mol^−1^ and 104.8 kcal mol^−1^, respectively, higher than those calculated for the DPPC membrane (Table 2). In addition, the energy difference between the two peptides was small, being 3 kcal mol^−1^ higher for the KAibG peptide, similar to the results obtained *via* SMD simulations. Furthermore, both KAibA and KAibG displayed a similar number of contacts with close molecules in the DPPC : DOPC : Chol membrane compared with those in the DPPC membrane (Fig. 4d and f), consistent with their calculated energies for internalization.

The KAibA secondary structure in the DPPC : DOPC : Chol membrane differs from that observed in the DPPC membrane (Fig. 2c). Here, it had a higher propensity toward α- and 3_10_-helices than for turns and bends, as in the DPPC membrane (Fig. 2a), although Ala6 and Lys7 still have a predisposition to adopt a turn structure. The helical structure was similar to that observed in the SMD simulations. It adopted an amphipathic helix while in contact with the PC groups of the upper membrane leaflet, with its Lys residues in contact with the PC groups and water molecules and its Aib and Ala residues oriented toward the membrane interior. Internalization began with its hydrophobic residues, and the Lys residues remained in contact with the PC groups of the outer leaflet, pulling them toward the inside of the membrane during internalization (Fig. 5g). When it approaches the PC groups of the lower leaflet, it tends to adopt a more disordered structure than that at the beginning of internalization. As for the DPPC membrane, the 2D histogram of internalization shows that KAibA had more open conformations than KAibG (Fig. 3c), and the main internalization occurred when the distance from the N-terminus to the C-terminus was 8 to 10 Å in the helical conformation (Fig. 3c). In the DPPC : DOPC : Chol membrane, KAibG presented a higher propensity to adopt a secondary structure than KAibA (Fig. 2), similar to what was observed in the DPPC membrane. In the DPPC membrane, KAibG had a strong tendency to form a close compact structure composed of bends and turn. Here, the peptide displayed less tendency to adopt these secondary structures, while Aib6, Gly6, and Lys7 had a higher tendency to adopt a 3_10_-helix structure, especially when approaching the PC groups of the lower leaflet (Fig. 3d). The KAibG C- and N-termini also remained at a short distance for a long time (Fig. 3d), unlike the structure in the DPPC membrane, where the C-terminus does not come into contact with Gly3, and thus, the folding was not very compact.

In contrast to the DPPC membrane, the DPPC : DOPC : Chol membrane did not undergo membrane thinning before peptide internalization (Fig. S10†). Moreover, the PC groups of the upper and lower leaflets did not directly come into contact with one another (Fig. S10†). A water channel was transiently formed, but it rapidly closed once the peptides crossed the membrane. The hydrophobic residues drove peptide internalization to the lower leaflet, with Lys oriented toward the PC of the upper membrane layer. However, the PC groups were not dragged or pushed toward the lower leaflet by the peptides. Only when the peptide reached the PC groups of the lower leaflet did one Lys residue transition from the upper PC groups to come into contact with those in the lower leaflet, and Lys did not try to reach the PC groups of the lower leaflet before. These results suggest that the higher energies of the DPPC : DOPC : Chol membrane could be explained by a combination of these factors. In addition, it was reported that peptides bind and internalize less efficiently in membranes with cholesterol.^68^

### Comparison between BP100 and R_9_ for the KAibA and KAibG peptides

KAibA and KAibG peptide internalization was compared with that of two other peptides with cell penetration abilities, BP100 and R_9_,^2,3,5,28^ together with R_9_ composed exclusively of d-amino acids, d-R_9_. The use of a chiral peptide allowed the study of whether the stereoisomer peptide changes peptide internalization and comes into contact with the membrane. Due to the computational resources available, these simulations were performed using ABMD with a faster bias deposition (10 ps) and higher resolution of the bias than the previous simulations with KAibA and KAibG. However, to achieve more comparable results, the simulations with KAibA and KAibG were repeated under the same conditions as those used for BP100 and R_9_.

Of all the studied peptides, KAibA and KAibG showed lower internalization energies in both membranes (Table 3, Fig. S10–S11†). The energies calculated here were higher than those calculated using a slower deposition bias (Table 2), which was expected since the peptides had less time to adapt to their surroundings. Interestingly, however, the same general trends were observed: the energies of the DPPC membrane were smaller than those of the DPPC : DOPC : Chol membrane; KAibG in the DPPC membrane showed the lowest energy (84.5 kcal mol^−1^), and there was a small difference between the energies of these peptides in the DPPC : DOPC : Chol membrane. The secondary structural propensity analysis of KAibA and KAibG (Fig. S14†) agrees almost perfectly with those seen with the slower bias deposition (Fig. 2), and the landscapes for internalization displayed similar shapes and minima (Fig. 3, S12 and S13†). This suggests that the energy values calculated here were due to using a deposition bias and high resolution that led to an overestimation of the energies, but the secondary structures and internalization observed in these simulations agree qualitatively. BP100 and R_9_ showed very high energies for internalization in both membranes, although for the DPPC membrane, the values were slightly lower (Table 3, Fig. S10–S11†).

**Table 3:** Energies for the internalization of peptides into the DPPC membrane and DPPC : COPC : Chol membrane obtained by ABMD with a higher bias deposition

Membrane	Peptide	Δ*G* (kcal mol^−1^)
DPPC	KAibA	91.9
DPPC : DOPC : Chol	KAibA	139.4
DPPC	KAibG	84.5
DPPC : DOPC : Chol	KAibG	144.9
DPPC	BP100	172.8
DPPC : DOPC : Chol	BP100	188.5
DPPC	R_9_	206.7
DPPC : DOPC : Chol	R_9_	226.9
DPPC	d-R_9_	240.9
DPPC : DOPC : Chol	d-R_9_	217.6

**Fig. 6:**
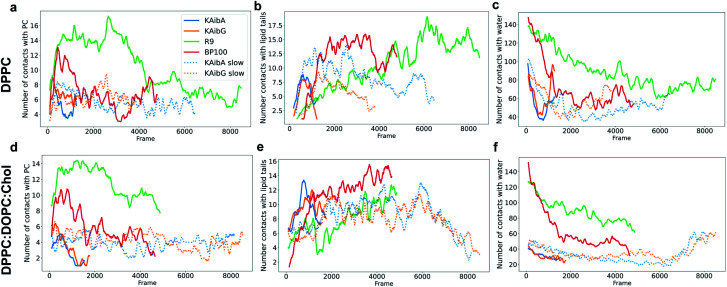
Number of contacts (molecules located at less than 3.5 Å) between the peptides and DPPC (top row) or DPPC : DOPC : Chol (bottom row) membrane molecules. Contacts between peptides and phosphatidylcholine (PC) (a, d), lipid tails (b, e) or water (c, f) over the simulation time. The lipid tails are palmitoyl groups for the DPPC membrane and palmitoyl plus oleoyl for the DPPC : DOPC : Chol membrane. The lines indicate a rolling average over 100 data points for the KAibA (blue), KAibG (orange), R_9_ (green) and BP100 (red) peptides. KAibA and KAibG ABMD with slower bias deposition are plotted for comparison in blue and orange dashed lines, respectively.

BP100 is a cationic peptide reported to form an amphiphilic α-helix when in contact with negatively charged membranes.^69^ However, other investigations did not find that BP100 adopted helical conformations while in contact with the plasma membrane or liposomes composed of DPPC : DOPC : Chol,^32,70^ suggesting that it binds in an unstructured conformation. Previous simulation studies also found that the α-content of BP100 in the DPPC membrane was small, in agreement with our structural propensity results.^71^ We did not observe membrane thinning of the membranes with BP100; rather, we observed only bending. In the DPPC membrane, BP100 adopted a fold with its N- and C-termini close together, similar to KAibG. Its Lys residues remained in contact with the PC groups, making a high number of contacts with them and with water molecules (Fig. 6a and c). Then, it internalized some of its hydrophobic residues (specifically, Phe, Leu and Tyr) into the membrane, increasing the number of contacts with the lipid tails (Fig. 6b, S18c†). Its Lys residues remained in contact with the PC groups, pulling them inside the membrane, but this interaction was lost over time when the contact with the lipid hydrophobic tails increased (Fig. 6a and b). When embedded in the membrane, the N-terminus (with the first two residues exhibiting a positive +3 charge) remained in contact with the PC groups and disrupted the H-bond with the C-terminal Leu, leading to internalization into the hydrophobic lipid tails (Fig. S12c†). The first amino acid residue that reached the PC groups on the lower leaflet was Tyr, with Lys reaching the lower leaflet later. In the DPPC : DOPC : Chol membrane, BP100 adopted a more extended conformation, with the hydrophobic residues (Leu, Tyr and Phe) rapidly internalizing into the lipid tails (Fig. 6e) and Lys pushing the PC of the top leaflet toward the lower leaflet. As the peptide internalized, it adopted a more compact structure (Fig. S13c†), with its N- and C-termini closer together, and the final internalization was similar to that in the DPPC membrane, with the Tyr reaching the lower membrane leaflet. The high-energy membrane crossing for BP100 can be attributed to the absence of membrane thinning and the higher number of positively charged Lys residues. These factors allowed BP100 to interact with more PC groups than KAibA or KAibG, especially in the DPPC membrane (Fig. 6a, d, S18a and b†). Nonetheless, it is the peptide with more hydrophobic interactions with lipid tails (Fig. 6b, e, S18c, d†) because of its hydrophobic residues.

R_9_ displayed the largest energies for internalization of all the studied peptides (Table 3). This finding is in agreement with studies that show that nona-arginine peptides at low concentrations are not able to penetrate the lipid membrane but are absorbed in the outer leaflet at the bottom of the lipid head groups.^72,73^ Furthermore, it was reported that R_9_ can internalize only into membranes containing negatively charged lipids such as phosphatidylglycerol (PG), but it has no or little effect on the DPPC membrane^68,74^ and the presence of cholesterol in the membranes reduces its internalization.^68^

R_9_ pushed the PC groups of the upper leaflet inside the membrane and forced them to reach the lower leaflet in the block, shielding the positively charged peptides of the hydrophobic lipid tails at all times. This was reflected in the high number of PC groups (Fig. 6a,d and S18a, b†) and water molecules (Fig. 6c, f and S19†) close to the peptides, the high number of H-bonds (Fig. S16, Table S2†) and the relatively low number of contacts with lipid tails (Fig. 6b, e). Indeed, there have been reports showing that the cationic Arg residues of some peptides cause salt bridge complexation between the guanidinium Arg group and the lipid phosphate and are able to drag the anionic phosphate groups inside the hydrophobic membrane.^75,76^ The internalization of the PC groups induced a strong bending of the membrane, and the lipid tails of the lipids on the lower leaflet were pushed downward. Previously, in our simulations with other peptides, the lipid tails of the lower membrane bent toward the hydrophobic part of the membrane, allowing the passage of the peptides and creating a pore-like structure. R_9_ was pushed down almost without bending its tails, mainly because R_9_ has no hydrophobic residues which are able to come into contact the lipid tails. With R_9_, the PC groups of the upper leaflet were at the forefront of the internalization process. R_9_ in the DPPC membrane adopted a compact structure to reach the lower leaflet of the membrane (Fig. S12d†), with Lys oriented toward the PC groups and the upper leaflet and the main chain of the peptide oriented toward the lipid tails. However, in the DPPC : DOPC : Chol membrane, we did not observe this compact structure, with the N-terminus farther away (Fig. S13d†). Nevertheless, R_9_ pulled the PC groups of the outer leaflet to the lower leaflet, forming a water pore, similar to that observed in the DPPC membrane with KAibA and KAibG (Fig. S8†). Arg-rich peptides have been shown to be able to form or nucleate toroidal pores,^75,77^ but the creation of such pores is thought to be a cooperative event involving several peptides.

### Effect of chirality on the internalization of the peptide

d-R_9_ presented little secondary structural propensity in the DPPC membrane, in contrast to the other peptides, particularly R_9_, with only slight bends (Fig. S14†). This indicates that d-R_9_ internalized in a disordered state, with its arginine residues in contact with the PC of the upper leaflet of the membrane until the end of the simulation. The lack of secondary structures can account for the high energy needed for the peptide to cross the membrane, with this peptide showing the highest of the calculated values (Table 3). The landscapes of R_9_ and d-R_9_ in the DPPC membrane presented a similar energy minimum at an N-terminus–C-terminus distance of 14 Å (Fig. S12d, e†). However, R_9_ presented a smaller distance between its N- and C-termini when it was close to the lower membrane leaflet (Fig. S12d†). In contrast, d-R_9_ retained an N-terminus–C-terminus distance of at least 10 Å apart through most of the simulation (Fig. S12e†).

In contrast to the lack of secondary structures in the DPPC membrane, in the DPPC : DOPC : Chol membrane, d-R_9_ presents a strong α-helix conformation propensity during all internalization processes (Fig. S15e†). The α-helical structure contributes to the lower internalization energy shown for d-R_9_ in the DPPC : DOPC : Chol membrane, facilitating a more compact structure and reducing the interaction of its arginine residues with the lipid tails (Fig. S17b†). This lower number of contacts with the lipid tails on the DPPC : DOPC : Chol membrane was also observed for R_9_ (Fig. 4), and there were small differences between R_9_ and its chiral peptide in terms of its interactions with the membrane.

We did not observe a significant increase in the internalization of d-R_9_ when compared to R_9_ in the simulations. Thus, the internalization improvement was probably caused not by a change in the internalization mechanism due to its chirality but by the intrinsic resistance to proteases, which effectively increased its active time and local concentrations.^6,20^

Due to computational limitations, we could simulate only a limited timescale, and we have studied the penetration of individual peptides into membranes. However, we note that all peptides studied are too short (9 to 12 residues) to span the bilayer thickness, especially if they adopt an α-helical conformation, without membrane bending, disruption or thinning (DPPC membrane).^69^ Moreover, the peptide:lipid ratio can affect peptide internalization, requiring a collaborative effect with other peptides to achieve peptide clustering, pore formation or membrane disruption.^69,72,73^ Thus, individual peptide internalization into the membrane is expected to be difficult. In addition, other factors, such as the lipid composition of the membrane, could affect the binding of the peptides to the membranes and their internalization. Some studies have shown that BP100, and particularly R_9_, bind more efficiently to anionic membranes with a higher content of negatively charged PG than PC^70,74^ and that cholesterol hinders their binding and translocation through the membrane.^68^ This is consistent with our current results, where all peptides internalized more effectively in the membrane without cholesterol (Tables 1–3), and can be explained by the observation that peptides caused disruption and thinning of the DPPC membrane. Furthermore, membrane domains rich in cholesterol are usually thicker and more rigid than regions poor in cholesterol, with cholesterol straightening the lipid fatty acid chains. This makes the membrane more viscous, slowing lipid diffusion movement,^78^ and some studies have shown that CPPs are internalized preferentially in the DPPC-rich domains of the DPPC : DOPC : Chol membrane.^32^ Unfortunately, we did not observe lipid domain formation on our DPPC : DOPC : Chol membrane due to the limited timescale of the simulations.^79^ The free-energy calculations and simulations of the internalization of the peptides under these conditions are challenging. Other limiting factors of the simulations could be the oversimplification of the membranes used, the accuracy of the force fields, insufficient sampling of the energy landscape due to its high degrees of freedom, the choice of poor or insufficient CVs to describe the energy path, or a combination of all of these factors.^80^ Due to their complexity, energy dependence, size and timescale, endocytosis mechanisms are not accessible with MD simulations, but energy-independent mechanisms can be modeled with MD techniques.^81^ Although the internalization mechanism is difficult to determine and the free energy is difficult to accurately calculate, the simulations can provide valuable insights into the translocation mechanisms of the different peptides for further engineering.

## Conclusions

KAibA and KAibG present the lowest energies for internalization in DPPC and DPPC : DOPC : Chol membranes compared with BP100 and R_9_, two CPPs widely used and known for their penetration abilities. The lower internalization energies of KAibG and KAibA are due to their compact amphipathic structures, which agrees with experimentally determined values in previous reports,^6^ and together with their lower energies, supports their efficient internalization abilities. They have an equilibrated and flexible amphipathic structure with 3 charged lysine residues that bind to the PC groups and hydrophobic (Aib and/or Ala) residues that can penetrate the hydrophobic membrane. These features allow them to orient Lys toward the upper PC groups, while the Aib residues facilitate internalization, making hydrophobic contacts with the lipid tails. In contrast, R_9_ presents the highest internalization energy, as expected, since it presented little penetration into zwitterionic membranes. BP100, with 5 polar residues, also struggles to detach its polar residues from the PC groups and internalize them into the hydrophobic lipid tails. These two peptides drag more PC groups and water molecules inside the membrane than KAibA/G, increasing their penetration energy. The different internalization of KAibA and KAibG in the membranes, causing membrane disruption and creating water pores in the DPPC membrane, suggests that the lipid composition is important for the efficient internalization of CPPs and agrees with the results that cholesterol hinders CPP internalization. Furthermore, the membrane thinning observed in the DPPC membrane before penetration helps reduce the internalization of the peptides. The current results provide significant insights into the internalization of the studied peptides and help to better understand how peptides can internalize into membranes.

## Author contributions

J. G.-D.: data curation, formal analysis, investigation, methodology, visualization, validation, writing – original draft, writing review & editing. K. N.: conceptualization, funding acquisition, project administration, validation, supervision, writing – review & editing.

## Conflicts of interest

There are no conflicts to declare.

## Supplementary Material

NA-004-D1NA00674F-s001
