# Supplementary material for: Molecular dynamics study of the internalization of cell-penetrating peptides containing unnatural amino acids across membranes
Source: Nanoscale Adv. 2021 Nov 10;4(2):397–407. doi: 10.1039/d1na00674f (PMC9419563; doi:10.1039/d1na00674f)
Supplement: NA-004-D1NA00674F-s001 [file NA-004-D1NA00674F-s001.pdf]

Electronic Supplementary Material (ESI) for Nanoscale Advances.
This journal is © The Royal Society of Chemistry 2021

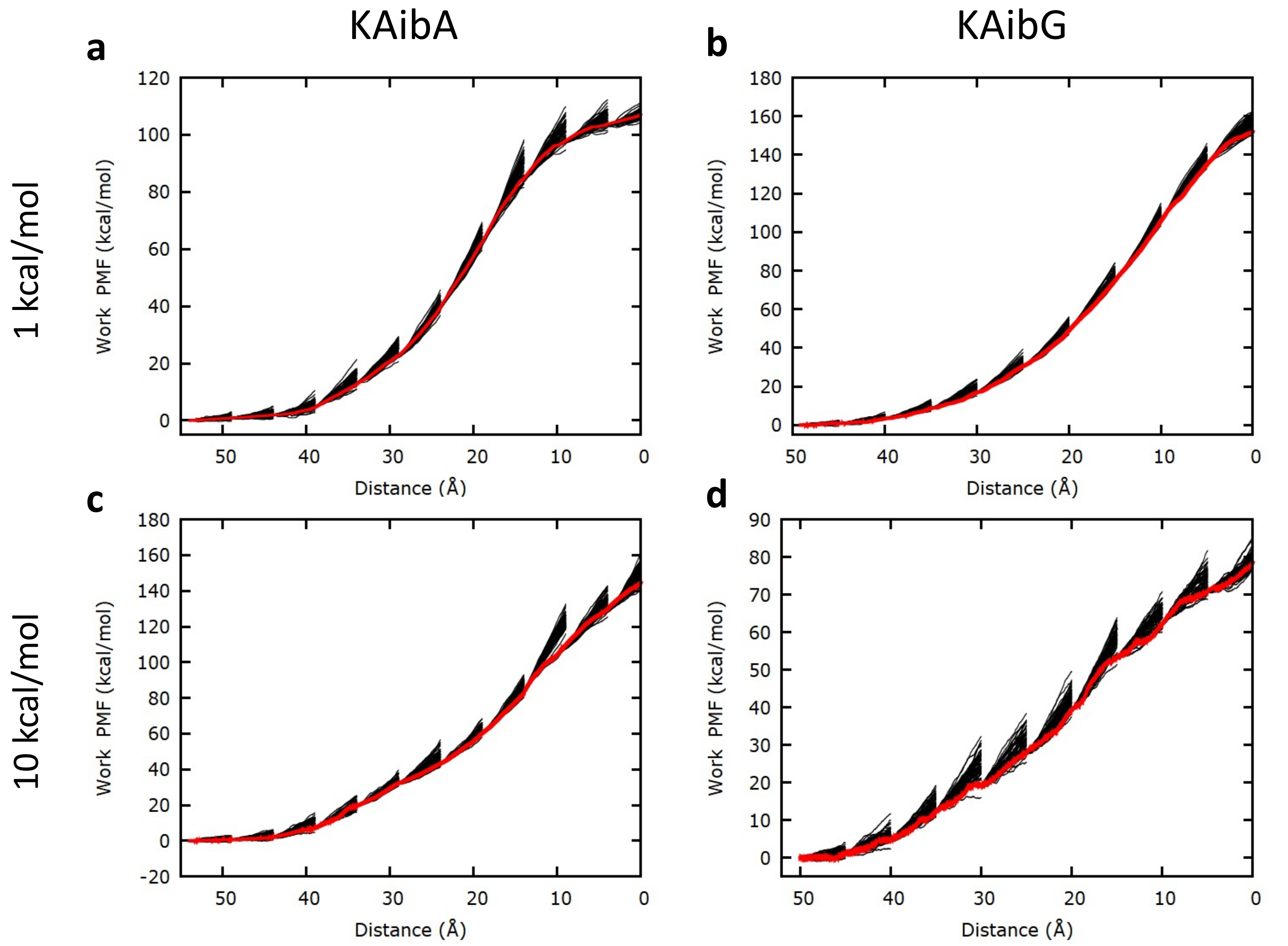

Figure S1. Potential of mean force obtain from the simulations of KAibA or KAibG peptides in a DPPC membrane using 1 kcal/mol (a,b) or 10 kcal/mol (c,d) harmonic force. Black lines are the work distributions obtained for individual simulations on each stage. Red line is the Potential of Mean force obtained with the Jarzynski equality.

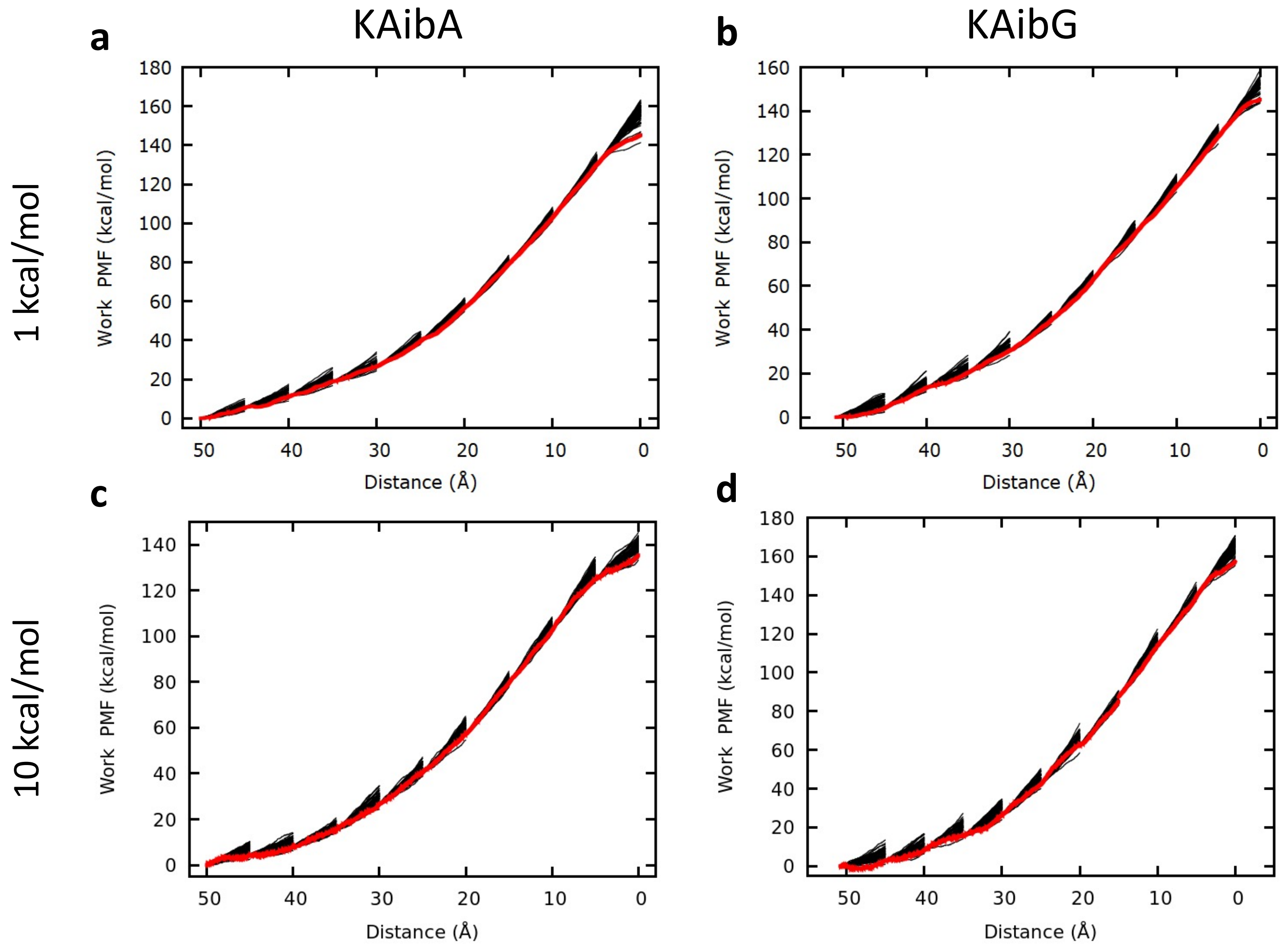

Figure S2. Potential of mean force obtain from the simulations of KAibA or KAibG peptides in a DPPC:DOPC:Chol membrane using 1 kcal/mol (a,b) or 10 kcal/mol (c,d) harmonic force. Black lines are the work distributions obtained for individual simulations on each stage. Red line is the Potential of Mean force obtained with the Jarzynski equality

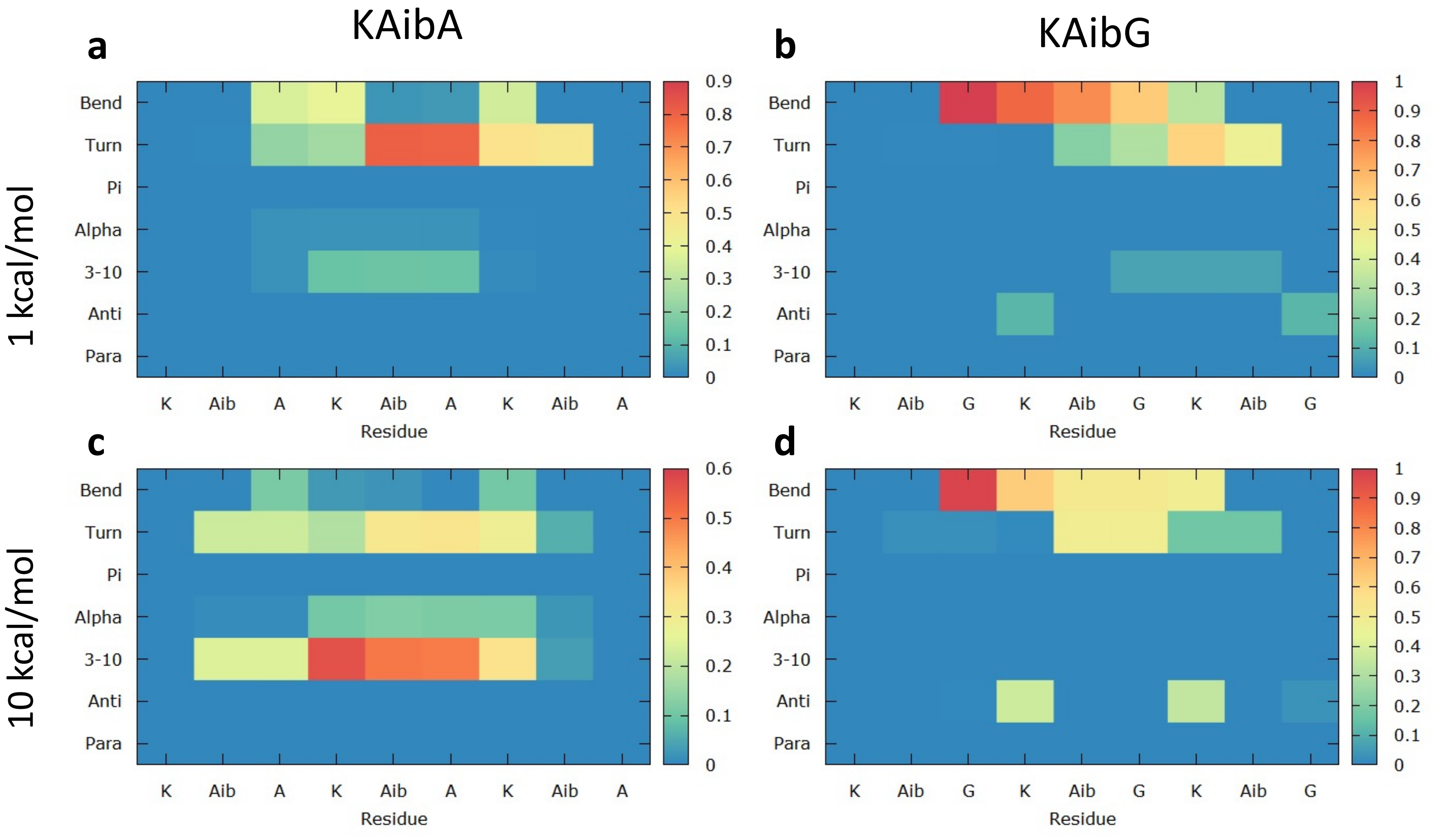

Figure S3. Average structural propensities from the simulations of KAibA or KAibG peptides in a DPPC membrane using 1 kcal/mol (a,b) or 10 kcal/mol (c,d) harmonic force. The propensities are calculated over all frames for each amino acid residue. Para; parallel β-sheet. Anti; anti-parallel β-sheet. 3-10; 3-10 helix. Alpha; α-helix. Pi; Pi (3-14) helix. Color bar indicates propensity.

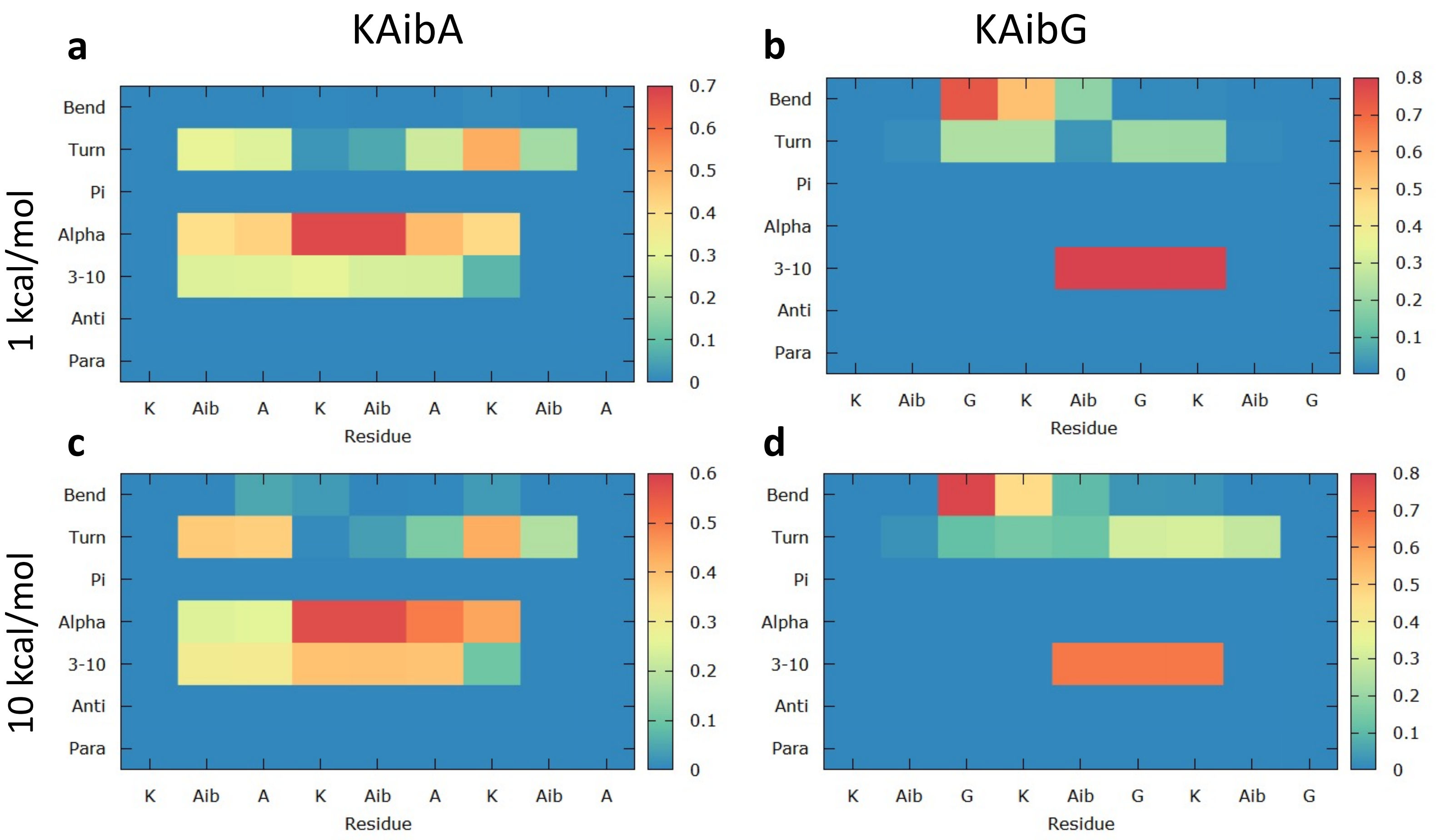

Figure S4. Average structural propensities from the simulations of KAibA or KAibG peptides in a DPPC:DOPC:Chol membrane using 1 kcal/mol (a,b) or 10 kcal/mol (c,d) harmonic force. The propensities are calculated over all frames for each amino acid residue. Para; parallel β-sheet. Anti; anti-parallel β-sheet. 3-10; 3-10 helix. Alpha; α-helix. Pi; Pi (3-14) helix. Color bar indicates propensity.

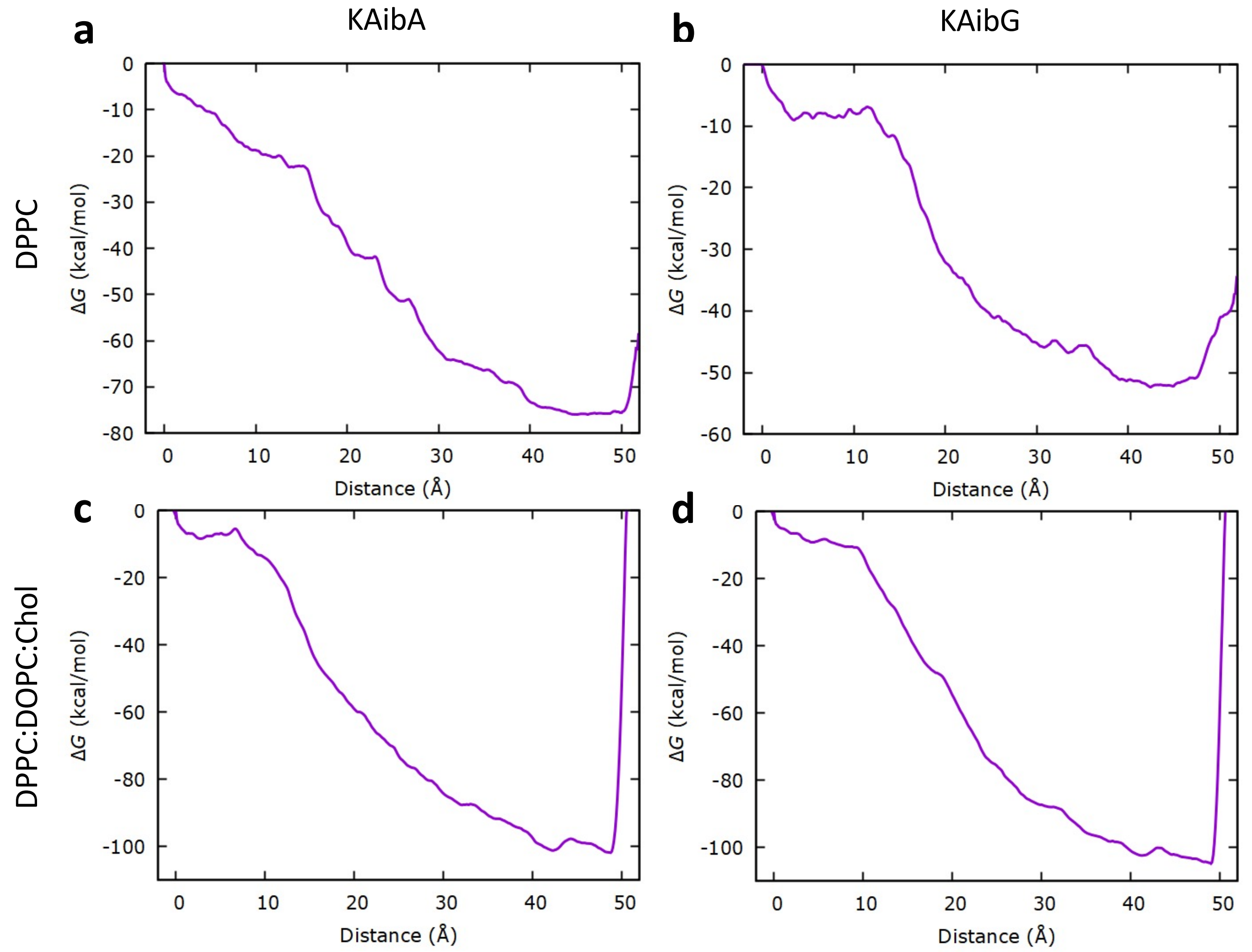

Figure S5. Energies obtained for the internalization of KAibA and KAibG peptides into DPPC (a,b) and DPPC:COPC:Chol (c,d) membrane.

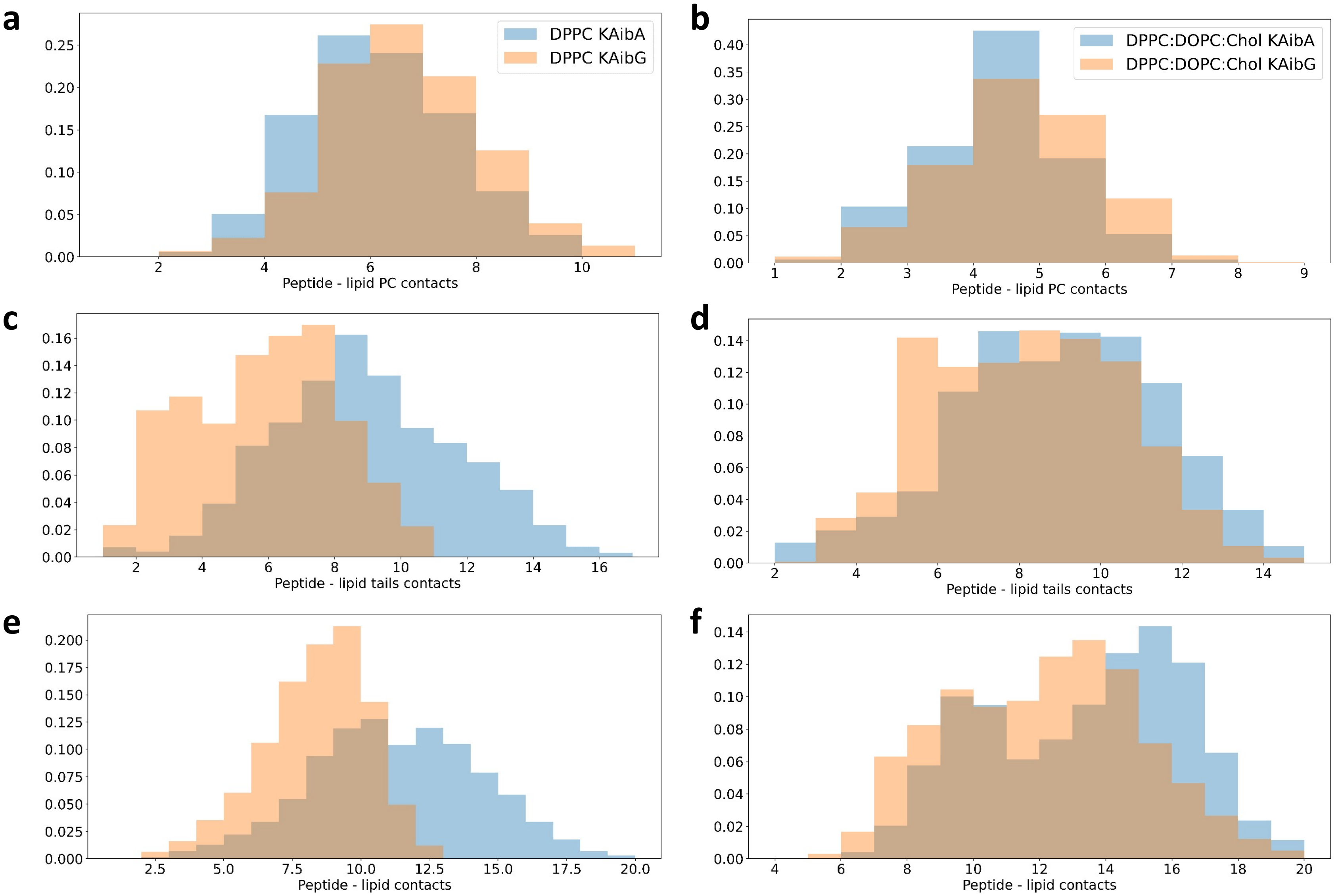

Figure S6. Normalized number of contacts (a contact is defined by a molecule located at less of 3.5 Å distance) between KAibA or KAibG with lipids over the simulation time in the ABMD simulations. (**a,c,e**) Contacts with lipids in the DPPC membrane **(b,d,f**) and in the DPPC:DOPC:Chol membrane. (**a,b**) Contact with phospatidylcholine (PC) atoms. (**c,d**) Contact with lipid tails (dipalmytoil (PA) and dioleoyl (OL)). **(e,f)** All peptide – lipid contacts, including cholesterol for DPPC:DOPC:Chol membrane (**f**).

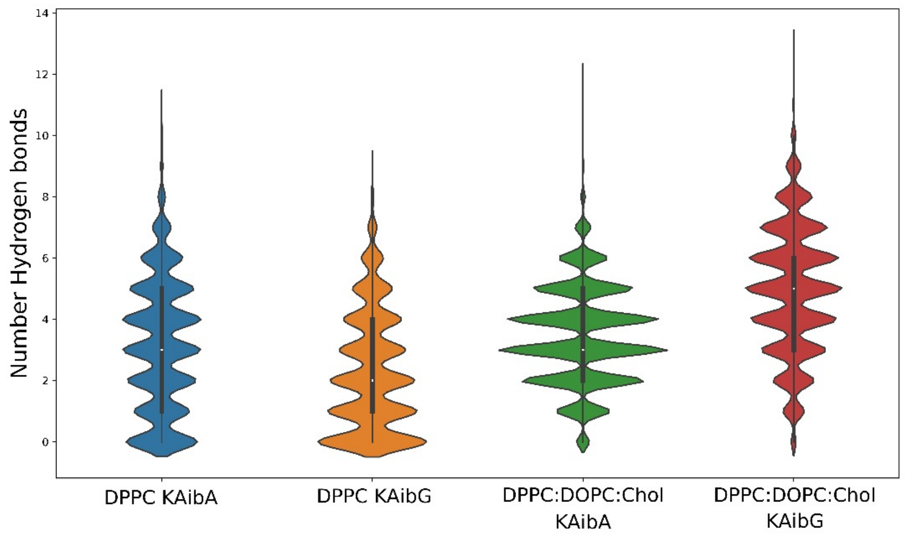

Figure S7: Hydrogen bond analysis for the ABMD simulations of KAibA and KAibG in DPPC and DPPC:DOPC:Chol membranes, showing the probability density of each H-bond value.

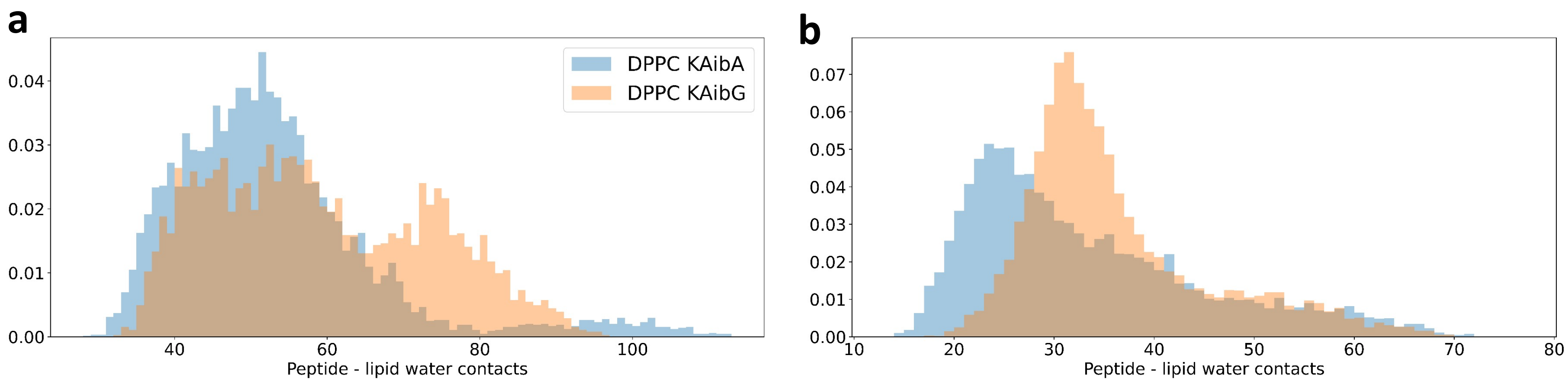

Figure S8. Normalized number of contacts between KAibA or KAibG with water molecules located at less of 3.5 Å in the DPPC (**a**) and DPPC:DOPC:Chol membrane (**b**).

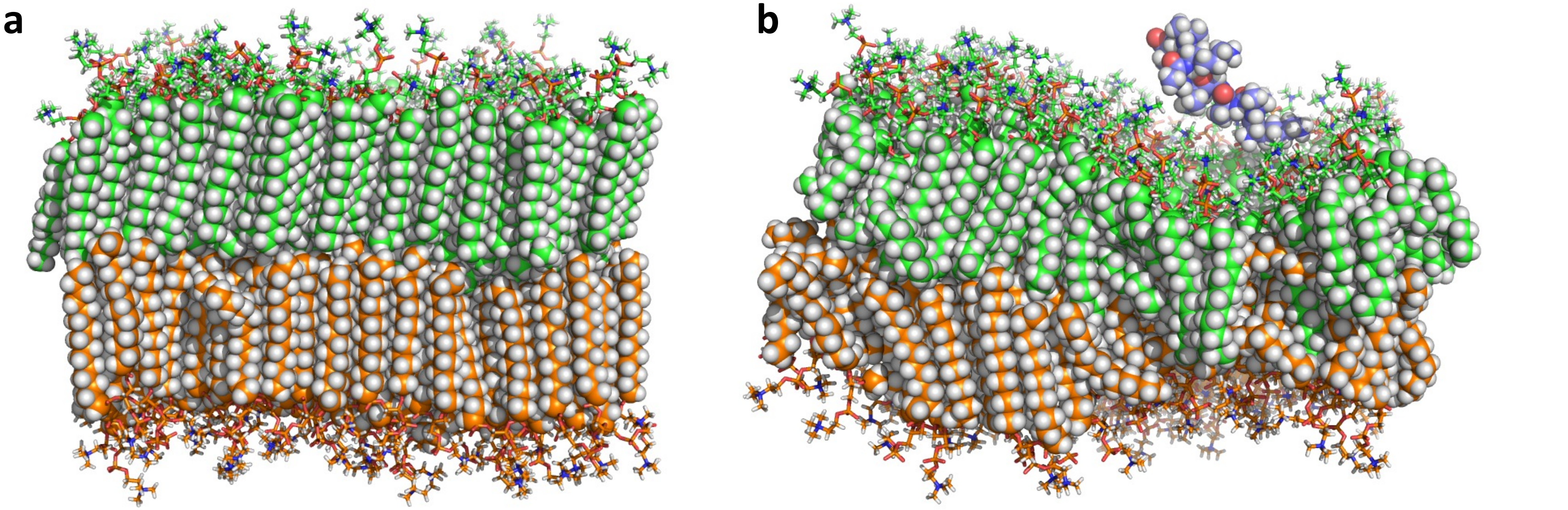

Figure S9. Molecular dynamics simulations of the structure of DPPC membrane with and without CPP**. (a)** Equilibrated DPPC membrane without CPP. **(b)** DPPC membrane thinning induced by the presence of CPP before penetration. Lipids tails (palmitic acid) and peptide atoms are displayed as spheres representation, phophatidylcholine groups of lipids are shown in sticks. Carbon atoms of the upper leaflet, lower leaflet and peptide are displayed in green, orange and blue, respectively.

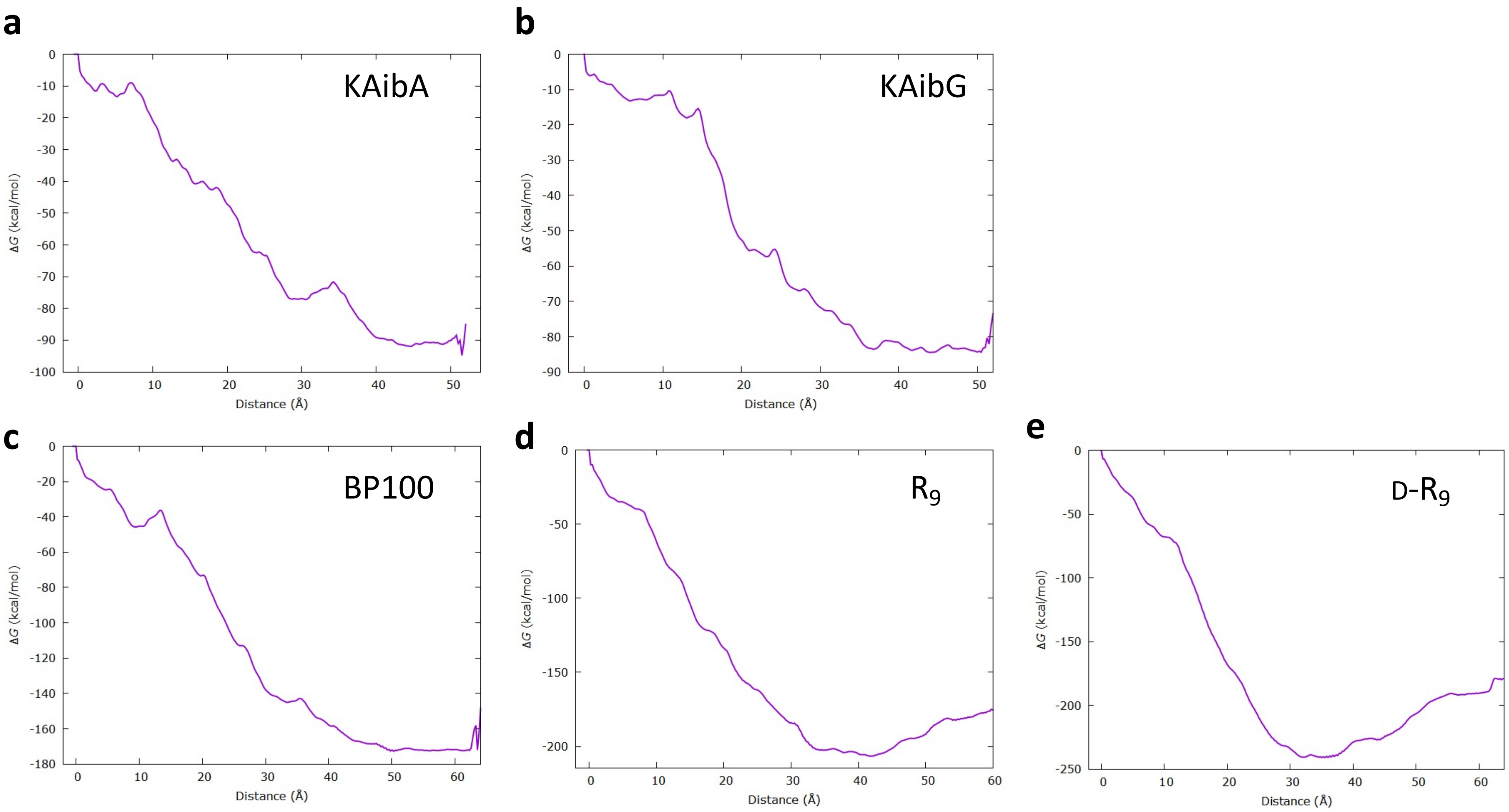

Figure S10. Potentials of mean force for the internalization of the studied peptides (a) KAibA, (b) KAibG, (c) BP100, (d) R_9 and (e) D-R_9 in the DPPC membrane.

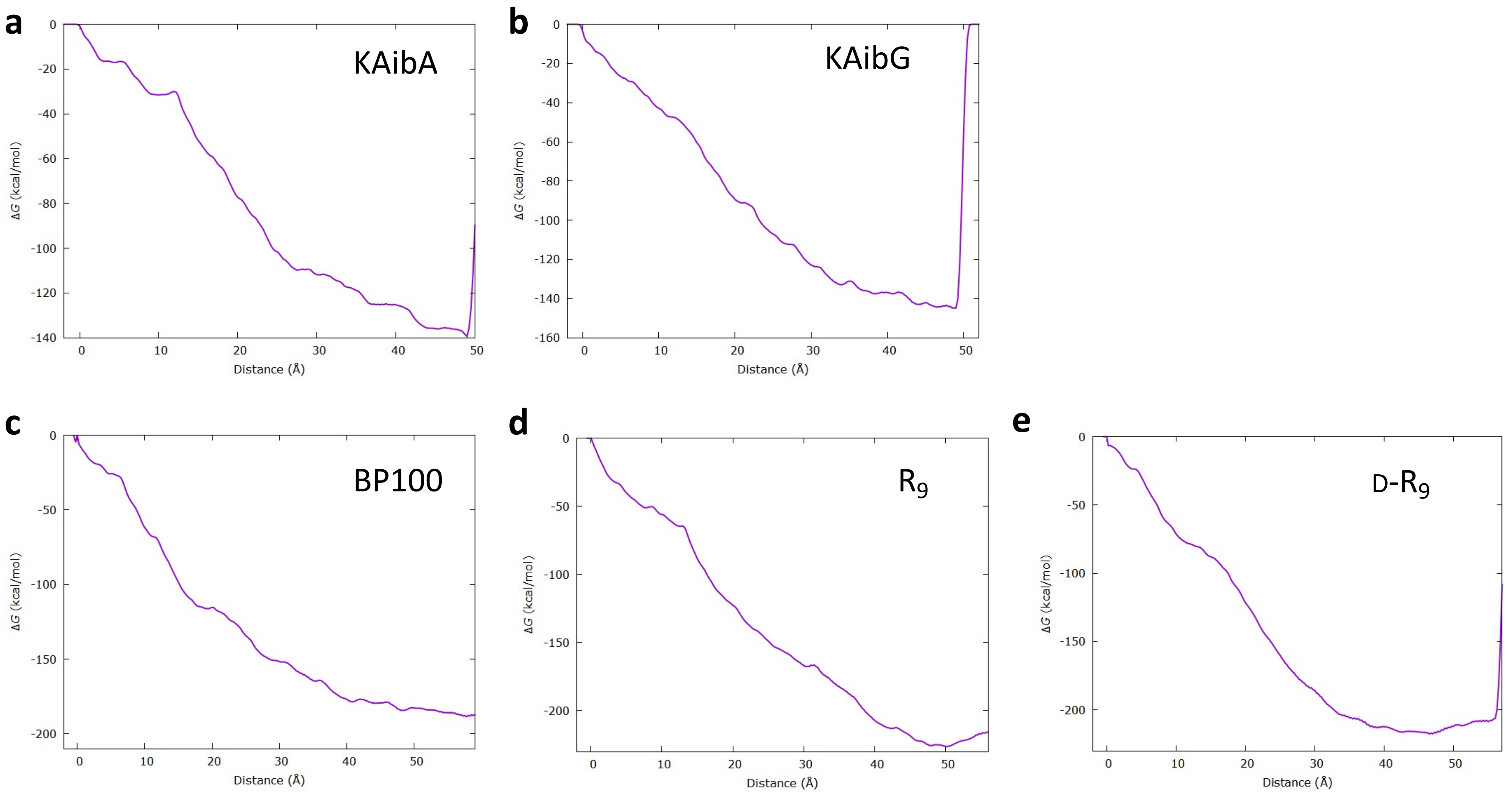

Figure S11. Potentials of mean force for the internalization of the studied peptides (a) KAibA, (b) KAibG, (c) BP100 (d) R_9 and (e) D-R_9 in the DPPC:DOPC:Chol membrane.

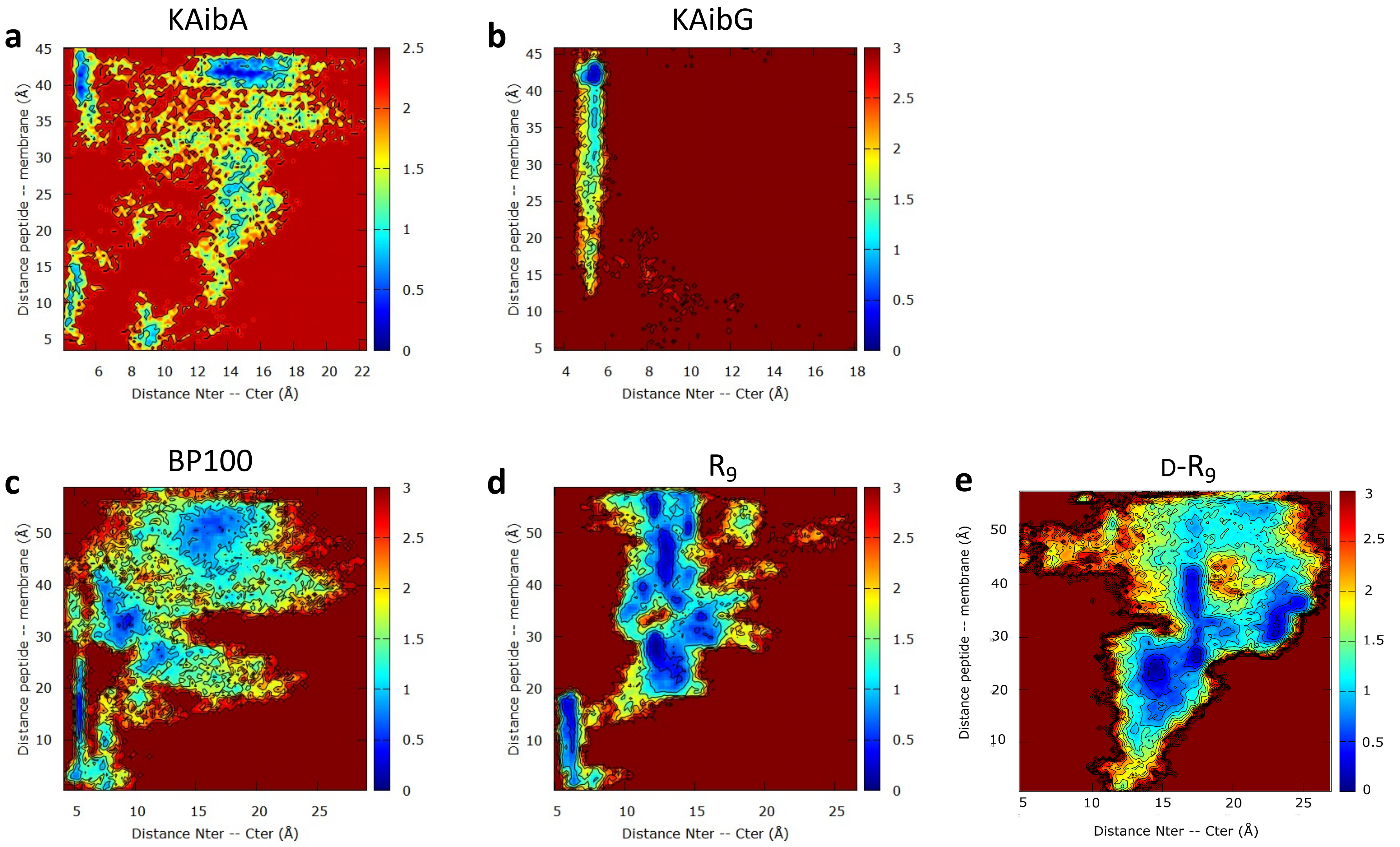

Figure S12. Two dimension histograms for peptides in the DPPC membrane obtained from ABMD simulations, using the COM distance between the α-carbon of the N-terminus and the α-carbon of the C-terminus amino acid of peptides, and the COM distance of the peptides to the membrane used as CV. (a) KAibA, (b) KAibG, (c) BP100 (d) R_9 and (e) D-R_9. The free energy (kcal/mol) is an estimation based on the bin population at 300 K all frames for each amino acid residue.

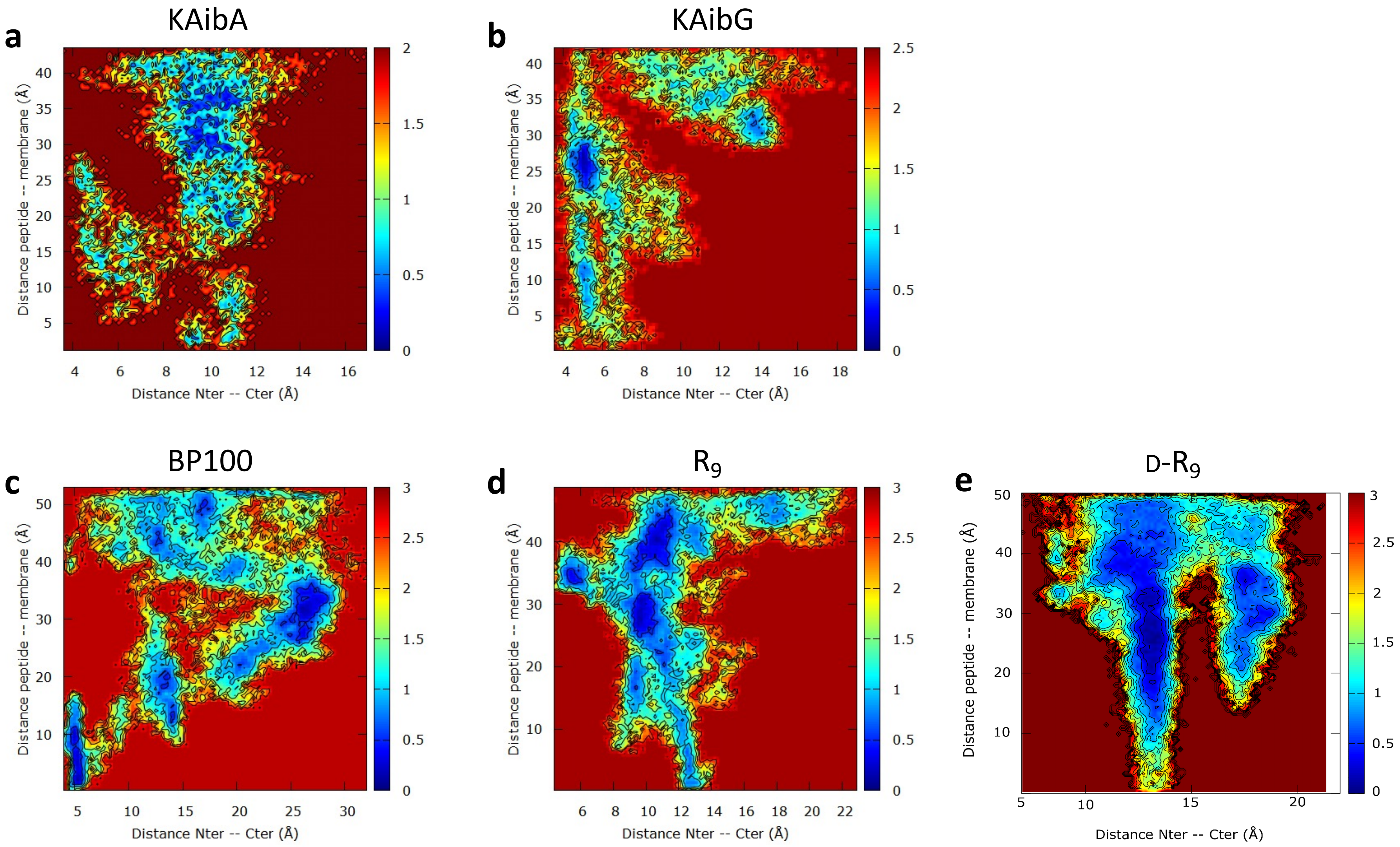

Figure S13. Two dimension histograms for peptides in the DPPC:DOPC:Chol membrane obtained from ABMD simulations, using the COM distance between the α-carbon of the N-terminus and the α-carbon of the C-terminus amino acid of peptides, and the COM distance of the peptides to the membrane used as CV. (a) KAibA, (b) KAibG, (c) BP100 (d) R_9 and (e) D-R_9. The free energy (kcal/mol) is an estimation based on the bin population at 300 K all frames for each amino acid residue.

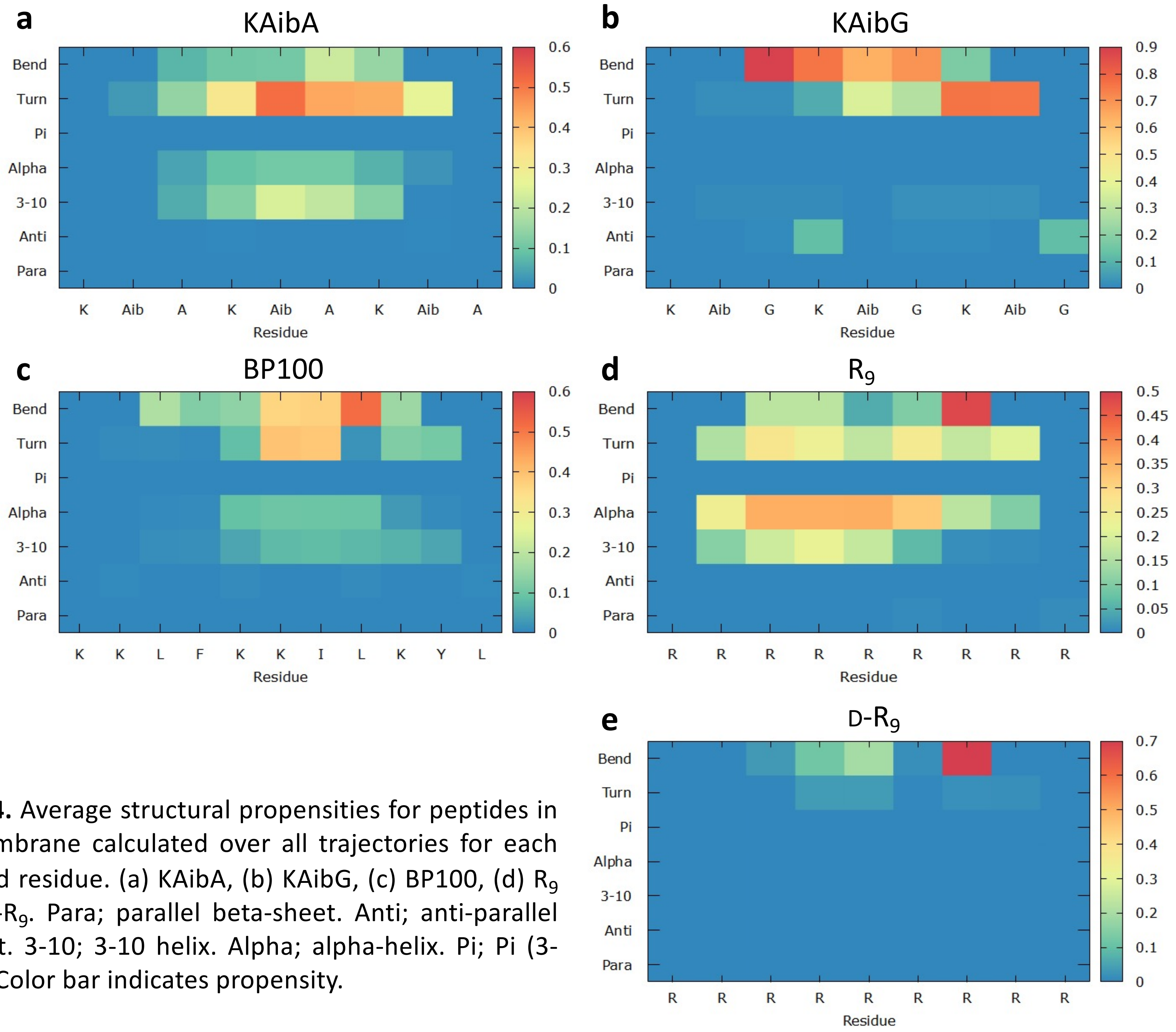

Figure S14. Average structural propensities for peptides in DPPC membrane calculated over all trajectories for each amino acid residue. (a) KAibA, (b) KAibG, (c) BP100, (d) R_9 and (e) D-R_9. Para; parallel beta-sheet. Anti; anti-parallel beta-sheet. 3-10; 3-10 helix. Alpha; alpha-helix. Pi; Pi (3-14) helix. Color bar indicates propensity.

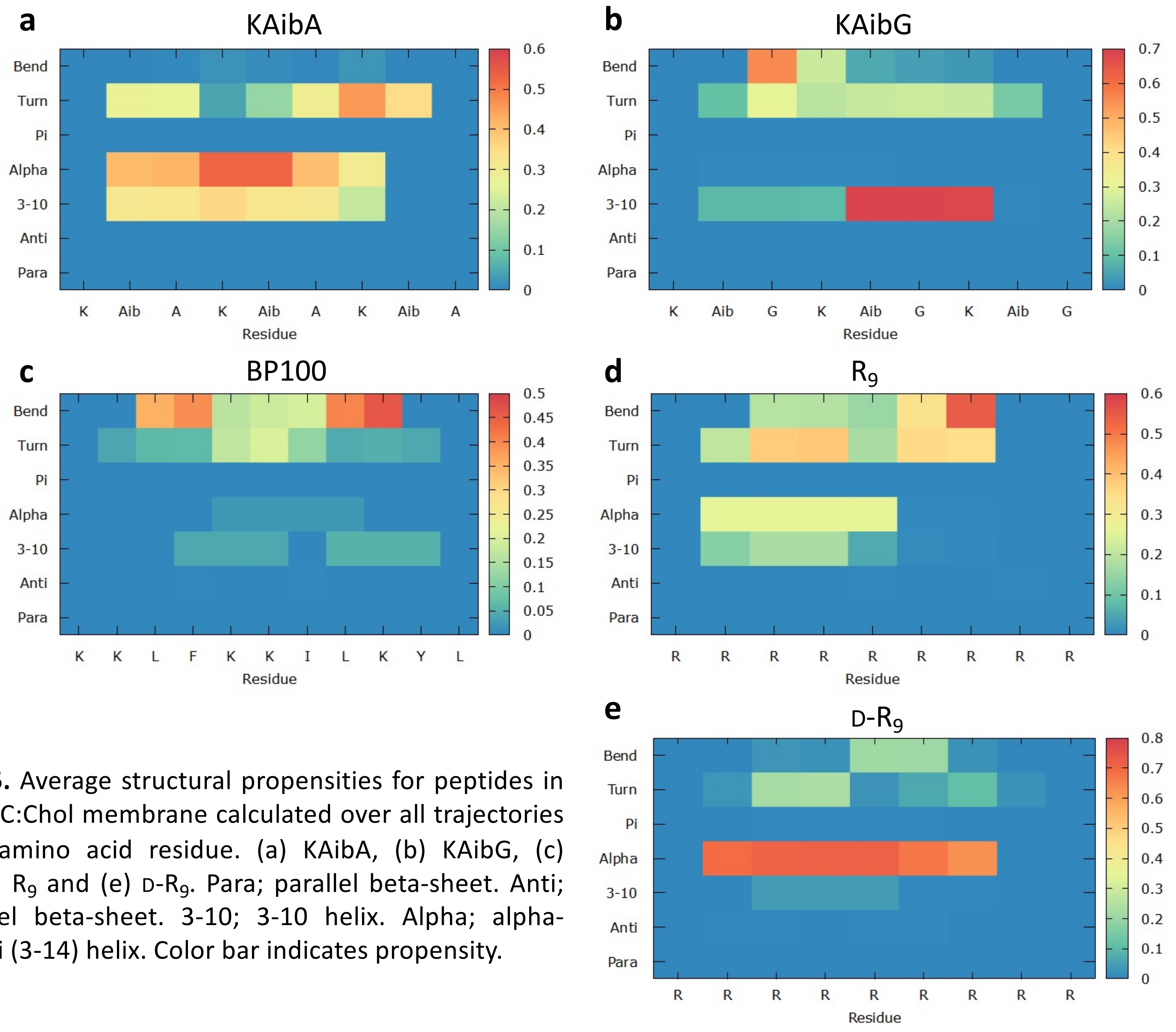

Figure S15. Average structural propensities for peptides in DPPC:DOPC:Chol membrane calculated over all trajectories for each amino acid residue. (a) KAibA, (b) KAibG, (c) BP100, (d) R_9 and (e) D-R_9. Para; parallel beta-sheet. Anti; anti-parallel beta-sheet. 3-10; 3-10 helix. Alpha; alpha-helix. Pi; Pi (3-14) helix. Color bar indicates propensity.

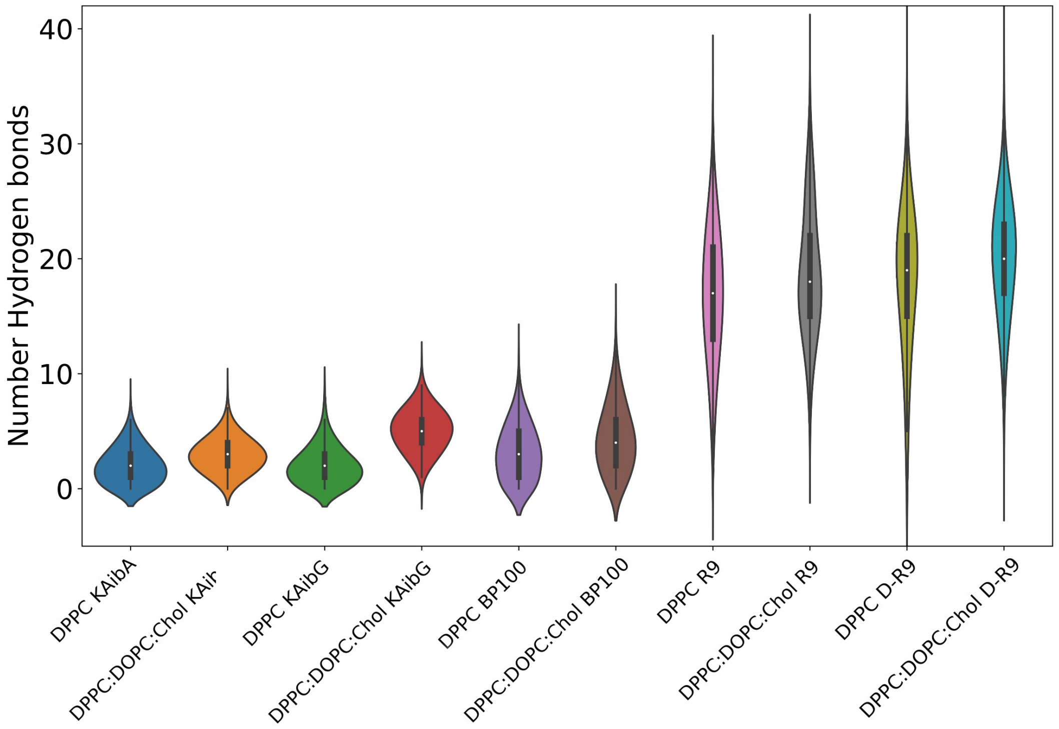

Figure S16: Hydrogen bond analysis for the ABMD simulations of KAibA, KAibG, BP100 and R_9 and D-R_9 in DPPC and DPPC:DOPC:Chol membranes, showing the probability density of each H-bond value.

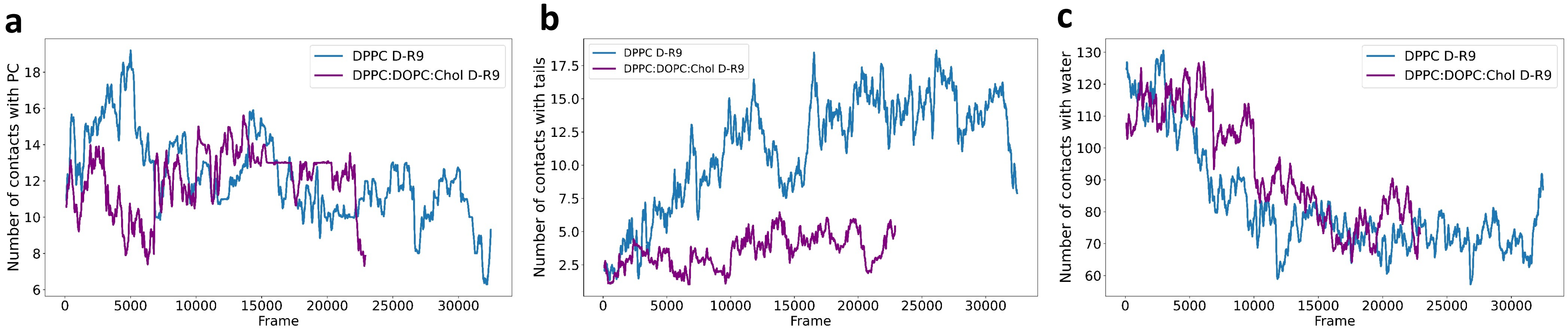

Figure S17. Number of contacts between the D-R_9 peptides and DPPC (blue line) or DPPC:DOPC:Chol membrane (purple line) over the simulation time. **(a)** Contacts between peptides and phosphatidylcholine (PC), **(b)** lipid tails and **(c)** water. Lipid tails are palmitoyl groups for DPPC membrane, and palmitoyl and oleoyl for DPPC:DOPC:Chol membrane. Contacts are defined as the molecules being located at less of 3.5 Å distance. Lines are a rolling average over 100 data points.

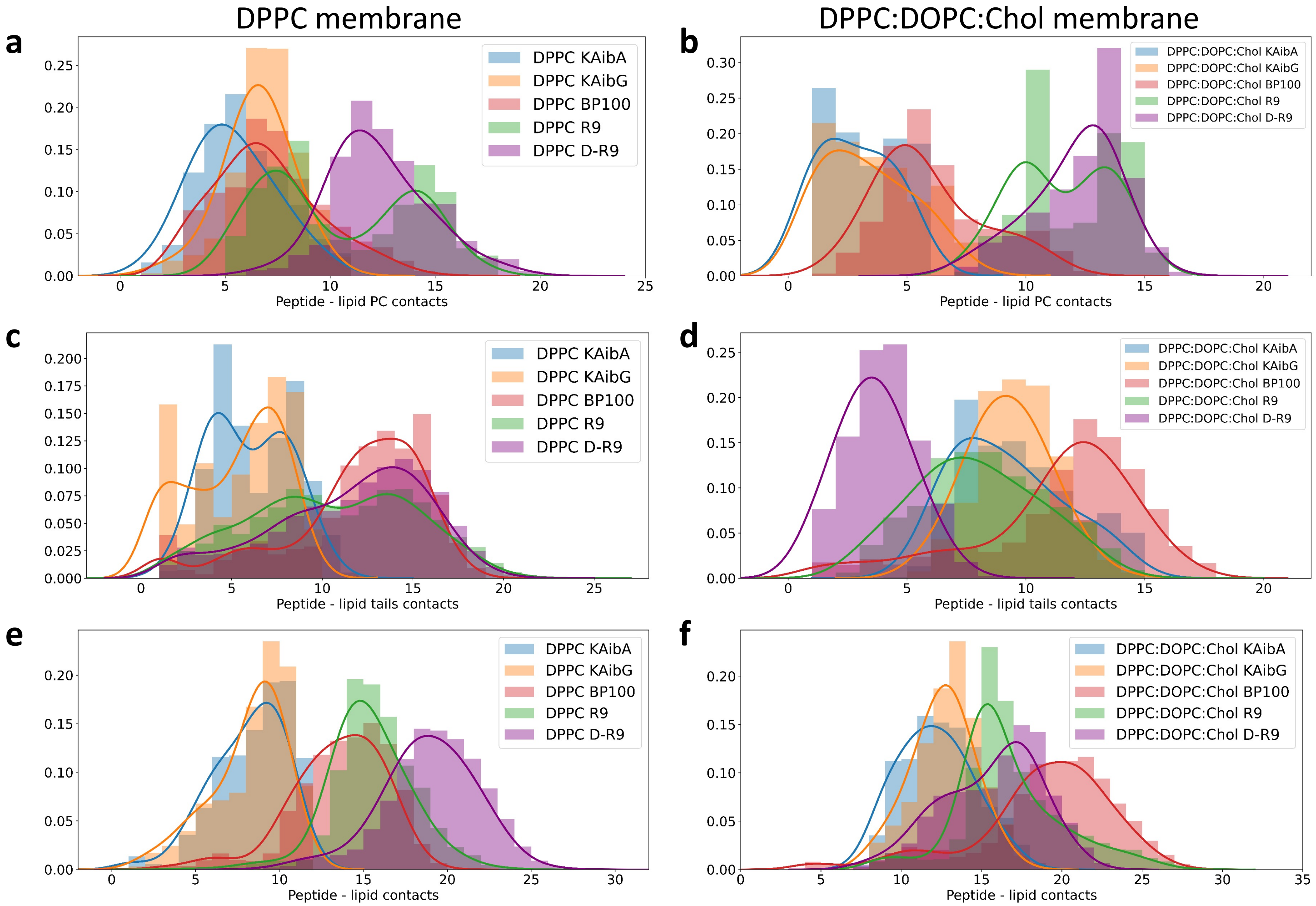

Figure S18. Normalized number of contacts between KAibA or KAibG with lipid or water molecules located at less of 3.5 Å distance over the simulation time. Contacts with lipids in the DPPC membrane (**a,c,e**) and in the DPPC:DOPC:Chol membrane **(b,d,f**). Contact with phospatidylcholine (PC) atoms (**a,b**). Contact with lipid tails (dipalmytoil (PA) and dioleoyl (OL)) (**c,d**). All peptide – lipid contacts **(e,f**) including cholesterol for DPPC:DOPC:Chol membrane (**f**).

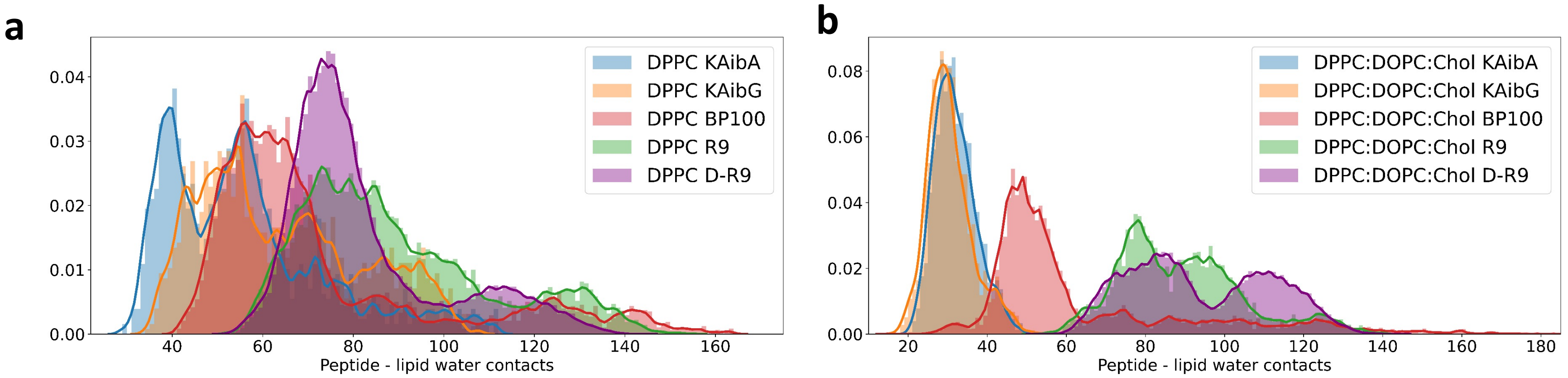

Figure S19. Normalized number of contacts between KAibA or KAibG with water molecules located at less of 3.5 Å in the DPPC **(a)** and DPPC:DOPC:Chol membrane **(b)**.

Table S1: Hydrogen bond analysis for the ABMD simulations of KAibA and KAibG in DPPC and DPPC:DOPC:Chol membranes.

Membrane	Peptide	Mean
DPPC	KAibA	3.13 ± 2.13
	KAibG	2.29 ± 1.93
DPPC:DOPC:Chol	KAibA	3.47 ± 1.58
	KAibG	4.95 ± 2.06

Table S2: Hydrogen bond analysis for the ABMD simulations of KAibA, KAibG, BP100 and R_9 and D-R_9 in DPPC and DPPC:DOPC:Chol membranes.

Membrane	Peptide	Mean
DPPC	KAibA	1.96 ± 1.52
DPPC:DOPC:Chol	KAibA	2.88 ± 1.44
DPPC	KAibG	1.94 ± 1.56
DPPC:DOPC:Chol	KAibG	5.03 ± 1.75
DPPC	BP100	3.07 ± 2.29
DPPC:DOPC:Chol	BP100	4.30 ± 2.78
DPPC	R_9	16.83 ± 5.43
DPPC:DOPC:Chol	R_9	18.75 ± 5.24
DPPC	D-R_9	18.11 ± 5.88
DPPC:DOPC:Chol	D-R_9	20.09 ± 4.77
